# Multidimensional self-reported sleep health, cognitive decline, and risk of all-cause dementia: A population-based multi-cohort study

**DOI:** 10.1177/13872877261422263

**Published:** 2026-03-16

**Authors:** Sanne J. W. Hoepel, Nina Oryshkewych, Lisa L. Barnes, Meryl Butters, Daniel Buysse, M. Kamran Ikram, Andrew Lim, Frank J. Wolters, Lan Yu, Meredith L. Wallace, Annemarie I. Luik

**Affiliations:** 1 Department of Epidemiology, 6993 Erasmus MC University Medical Centre, Rotterdam, Netherlands; 2 University of Pittsburgh Medical Center, Pittsburgh, PA, USA; 3 207044 Rush Alzheimer's Disease Center, Rush University Medical Center, Chicago, IL, USA; 4 Department of Psychiatry, 6614 University of Pittsburgh, Pittsburgh, PA, USA; 5 Department of Neurology, Erasmus University Medical Centre, Rotterdam, Netherlands; 6 Department of Neurology, 7938 University of Toronto, Toronto, Ontario, Canada; 7 Department of Medicine, 6614 University of Pittsburgh, Pittsburgh, PA, USA; 8 Trimbos Institute - The Netherlands Institute of Mental Health and Addiction, Utrecht, The Netherlands

**Keywords:** Alzheimer's disease, cluster analysis, cognitive function, cohort studies, dementia, sleep, sleep duration, sleep quality

## Abstract

**Background:**

Considering the multidimensional nature of self-report sleep health may improve identification of those at risk of accelerated cognitive decline and dementia.

**Objective:**

We compared how composite measures of multidimensional sleep health relate to cognitive performance and the risk of dementia over time in older adults.

**Methods:**

Self-reported indicators of sleep health domains (satisfaction, alertness, timing, efficiency, and duration) were measured in 7892 Rotterdam Study (RS) participants (mean ± SD age: 69.5 ± 8.9 years, 58.2% female) and 1601 Rush Memory and Aging Project and Minority Aging Research Project (MAP/MARS) participants (79.5 ± 7.9 years, 77.3% female). Sleep items were harmonized and used to derive a sleep health score (number of adverse sleep health items) and sleep health clusters (with latent class analysis). During follow-up, multiple cognitive tests were performed repeatedly and participants were followed for incident all-cause dementia. Relationships of sleep health with cognitive decline (linear mixed models) and risk of dementia (Cox proportional hazards models) were assessed in both samples, adjusting for covariates.

**Results:**

Three sleep health clusters were identified: average sleep, inefficient sleep, and poor sleep. During follow-up of 10.6 ± 4.5 years in RS and 5.3 ± 2.9 years in MAP/MARS, 1148 (14.5%) and 286 (19.8%) participants developed dementia, respectively. Multidimensional sleep health scores and clusters were not significantly associated with accelerated cognitive decline or the risk of dementia in either sample (Hazard Ratios [HRs] between 0.72–1.15).

**Conclusions:**

Findings suggest composite measures of self-reported multidimensional sleep health need refinement to be useful in identifying older adults at risk of accelerated cognitive decline and dementia.

## Introduction

Sleep health is multidimensional and can be characterized across domains including regularity, satisfaction, alertness, timing, efficiency, and duration.^1^ Poor sleep health has been shown to predict mortality, frailty, and heart disease and to relate to mental health; and can be quantified as the number of self-reported poor sleep health items.^2–10^ Moreover, person-centered approaches such as clustering analysis can derive sleep health clusters that reflect common within-person patterns of multidimensional sleep health.^11–13^ These clusters can provide unique insight into the specific combinations of sleep health domains that are associated with health outcomes. Recently we identified self-report sleep health clusters that are generalizable across multiple cohorts of older adults and predict depressive symptoms.^13^

Accumulating evidence supports a bi-directional association between poor sleep health and neurodegeneration, meaning that poor sleep health is likely both a cause and a consequence of pathophysiological processes underlying cognitive decline and dementia.^14–22^ Pathways through which poor sleep may contribute to cognitive aging include increased neuro-inflammation, greater extracellular amyloid-β deposition, and disrupted neurogenesis.^14,17^ Vice versa, accumulating pathophysiological processes throughout the prodromal phase of dementia may affect sleep, for example via structural changes in sleep- and wake promoting nuclei.^23^ Poor sleep health thus has the potential to be an important early predictive and prognostic marker for accelerated cognitive decline and dementia.^14^ Sleep health might also be a potential target for prevention or disease-modifying interventions, especially given the high prevalence of poor sleep health amongst older adults.^24–26^ However, most evidence linking sleep to cognitive decline and dementia is limited to sleep disorders or specific aspects of sleep, such as sleep duration, whereas sleep health can only be captured fully by assessing the within-person pattern across multiple domains of sleep.^1^ Compared with objective measures of sleep, particularly polysomnography, self-reported sleep health across multiple domains is relatively easily assessed in routine clinical practice, making sleep health scores and clusters potentially important tools to identify those at risk of accelerated cognitive decline and dementia and inform targeted intervention and prevention strategies. However, evidence from previous studies assessing the association of sleep health scores and clusters with cognitive outcomes is inconclusive^27–29^ and it is unclear what measure of self-reported sleep health (i.e., sleep health score, sleep health cluster, or individual sleep items) is most informative to predict accelerated cognitive decline and dementia.

In the present study, we assessed the association of multidimensional sleep health with cognitive decline and the risk of dementia in population-based settings. We examined two common measures of multidimensional sleep health: a composite sleep health score quantifying ‘how many potentially adverse characteristics’ and sleep health cluster membership quantifying ‘which combinations of potentially adverse characteristics’. To enhance rigor and generalizability we performed a coordinated data analysis across two population-based samples, leveraging harmonized data from the SHARE (Sleep Harmonization and Replication) initiative.^30^

## Methods

### Study sample

**Figure 1.**
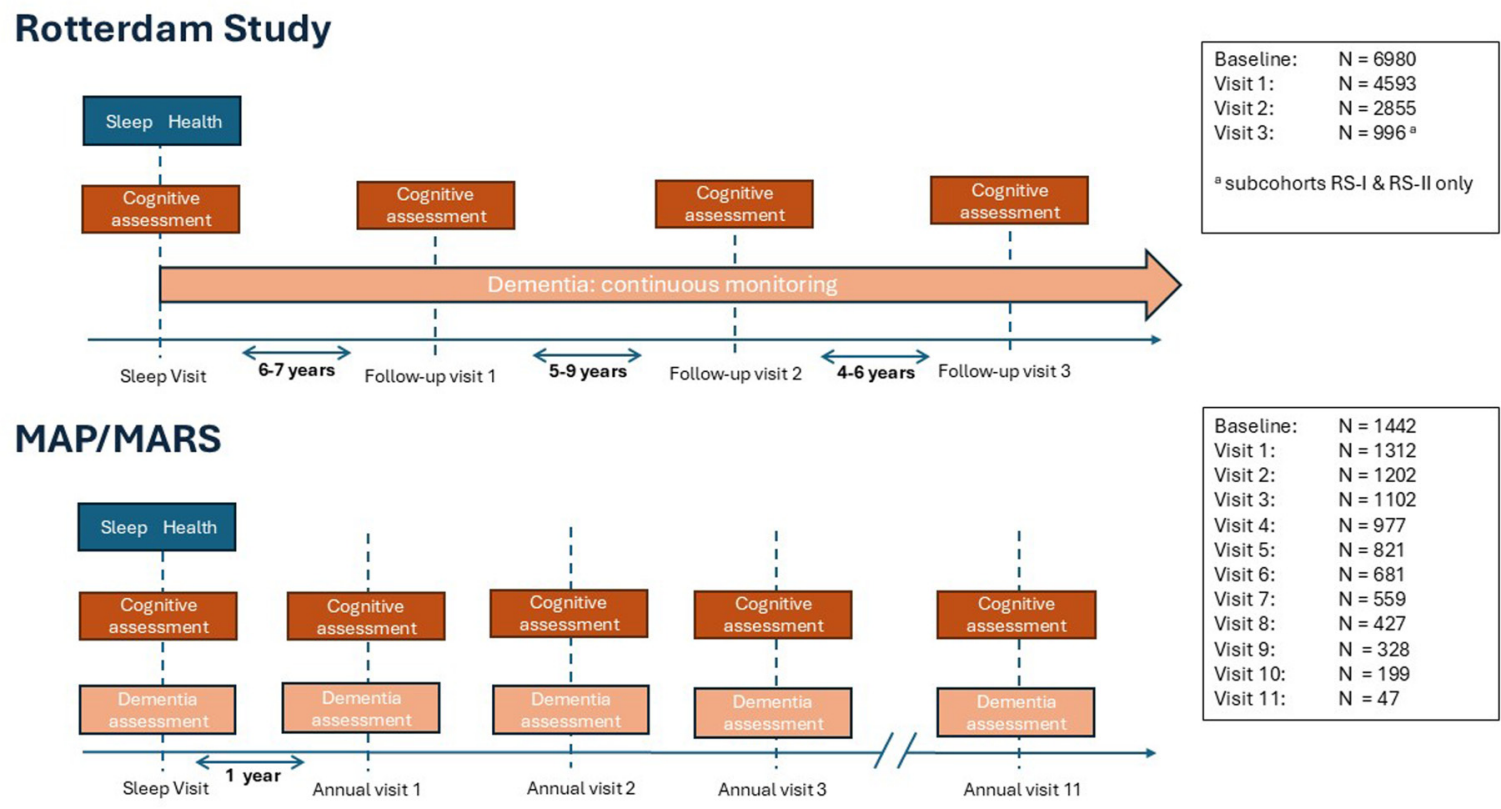
Study design.

*Rotterdam Study.* The Rotterdam Study (RS) is an ongoing prospective cohort of older adults in Rotterdam, the Netherlands, which was designed to investigate determinants and consequences of aging and age-related disease.^31^ The initial sleep visit in RS occurred between 2004 and 2011 when participants were first asked about their sleep during a home interview. Follow-up visits, including a home interview and visits to the research center, occurred every 4–9 years thereafter (Figure 1). Medical records were continuously followed for incident disease occurrence. In RS, 8365 participants who were > 55 years old participated in the baseline interview. We excluded 396 participants with incomplete data on sleep health or dementia at baseline, and 77 participants with prevalent dementia, leading to a final sample size of 7892 (94.3%) (Supplemental Figure 1). In models that examine how sleep health relates to trajectories of cognition, we additionally excluded 912 participants who did not participate in cognitive testing at baseline. Excluded participants were older, more often female and had lower education, more comorbidities, and poorer sleep health (Supplemental Table 1). Participants were followed until diagnosis of dementia, loss to follow-up, death, or January 1, 2020, whichever occurred first. Completeness of follow-up was high (i.e., 94.1% of potential person-years were observed).

*MAP and MARS.* The Rush Memory and Aging Project (MAP) and Rush Minority Aging Research Study (MARS) are longitudinal studies of adults over 65 that were designed to study cognitive decline and risk of Alzheimer's disease and other health outcomes.^32,33^ Participants from MAP and MARS were recruited from Chicago and broader northeastern Illinois in the United States through the Rush Alzheimer's Disease Center. These studies used highly overlapping methodologies and investigative teams and are analyzed as one sample in the current analysis. Measures of self-reported sleep and cognitive assessments are repeated annually (Figure 1); the initial sleep visit for each participant in the current study is the first observation year with complete data on self-report sleep health. In MAP/MARS, 1675 participants had complete sleep health data during the initial visit, of which 165 did not have complete data on dementia or were missing all cognitive tests at the initial sleep visit and 68 had prevalent dementia, leading to a final sample size of 1442 (85.7%) (Supplemental Figure 1). In MAP/MARS, excluded participants were similar to included participants for all sociodemographic and physical and mental health factors (Supplemental Table 2). Participants were followed until the first visit at which dementia was diagnosed, death, or loss to follow-up, whichever occurred first. In total, 62.1% of participants stopped participation more than 1 year before death. They were considered lost to follow-up and censored at the time of their last visit.

### Ethical approval

The Rotterdam Study has been approved by the Medical Ethics committee of the Erasmus MC (registration number MEC 02.1015) and by the Dutch Ministry of Health, Welfare and Sport (Population Screening Act WBO, license number 1071272-159521-PG) and has been entered into the Netherlands National Trial Register (NTR; www.trialregister.nl) and the WHO International Clinical Trials Registry Platform (ICTRP; www.who.int/ictrp/network/primary/en/) under shared catalog number NTR6831. Participants in all cohorts provided written informed consent to participate in longitudinal studies. MAP and MARS were approved by the Institutional Review Boards (IRBs) of their respective institutions. The SHARE Initiative received an exempt IRB declaration for data harmonization, aggregation, and analysis of secondary data from these cohorts.

### Measurements

*Sleep health.* In both samples, participants reported on sleep health at the initial sleep visit. For this study, sleep health was characterized by the five domains of Satisfaction, Alertness, Timing, Efficiency, and Duration (SATED).^1^ Consistent with our prior research,^13^ we selected comparable items across the samples for each of the SATED domains and categorized them as ‘good’ (scored as ‘0’) or ‘poor’ (scored as ‘1’). Cut-points were based on prior literature, clinical judgment, and the observed distribution and were consistent with our prior work.^12,13,30,34–36^ We defined poor sleep quality (Satisfaction) as a ‘fairly bad’ or ‘very bad’ sleep quality. High daytime sleepiness (Alertness) was defined as ‘somewhat’ or ‘very big’ daytime problems due to sleepiness. For midpoint of sleep (Timing), both an ‘early midpoint’ (<2AM) as well as ‘late midpoint’ (>4AM) were scored as poor.^34^ Sleep efficiency (Efficiency) is calculated as the percentage of time in bed spent asleep and poor efficiency was defined as sleep efficiency <85%^35^ . For nighttime sleep duration (Duration), both short sleep duration (<6 h) and long sleep duration (>8 h) were scored as poor.^36^

For each individual, a multidimensional sleep health score ranging from 0 to 5 was derived by summing the number of poor sleep items. Given that relatively few participants report 4 or 5 poor sleep health items, we generated the score with categories of 0, 1, 2, and 3–5 poor sleep health items. Next, we identified sleep health clusters by performing Latent Class Analysis (LCA) separately in both samples using the R poLCA package.^37^ We fit LCA models with 1 to 6 classes and calculated the Akaike's information criterion (AIC) and Bayesian information criterion (BIC) for each cluster solution, with lower values indicating improved model fit. The final number of clusters was selected based on 1) optimal model fit; 2) parsimony, i.e., we decided a priori not to consider clustering solutions that contained small clusters (relative size <5%) that are unlikely to be generalizable; 3) our previous work in the same samples.^13^ We calculated between-cluster Wasserstein distances to quantify the similarity of clusters within and across samples.^38^ We calculated Jaccard Indices to assess cluster stability and average posterior probabilities and entropy to assess separation, with higher values indicating better stability and separation.^39,40^

*Cognitive functioning.* In RS, cognition was assessed at every follow-up visit at the research center approximately every 4–6 years (Figure 1). In MAP and MARS, cognition was assessed at annual clinical visits. We selected cognitive tests in RS and MAP/MARS that matched as closely as possible. These were 1) a substitution task: the letter digit substitution task in RS and the symbol digits modality test in MAP/MARS; 2) a word learning task: the 15 word learning test – delayed recall in RS and the 10 word list test in MAP/MARS; and in both samples 3) the category fluency task; 4) the Stroop processing task; and 5) the Stroop interference task. These tests assess different aspects of cognitive functioning and are described in more detail in Supplemental Table 3 and elsewhere.^41,42^ Test scores were standardized into z-scores per sample to facilitate comparison of effect estimates within the sample. For each test, a higher score reflects better cognitive performance.

*Dementia.* In RS, participants were screened for dementia during each center visit and continuously monitored for dementia through linkage with medical records from general practitioners, the regional institute for outpatient mental health care, and nursing homes. Screening was based on the Mini-Mental State Examination and the Geriatric Mental Schedule organic level. Those with a Mini-Mental State Examination score less than 26 or Geriatric Mental Schedule score of more than 0 underwent further investigation and informant interview, including the Cambridge Examination for Mental Disorders of the Elderly. Additional medical information (e.g., clinical notes and neuro-imaging reports) was obtained from hospital records, if available. Whenever a potential case was identified, records were reviewed by research physicians and discussed in consensus meeting led by a consultant neurologist. The diagnosis of all-cause dementia was based on DSM-III-R criteria and clinical Alzheimer's disease was defined following NINCDS/ADRDA criteria.^43^

In MAP/MARS, clinical diagnosis of cognitive status is made at every visit to the research center based on a three stage process including computer scoring of cognitive tests, clinical judgment by a neuropsychologist, and a diagnostic classification by a clinician.^44^ Clinical diagnosis of dementia and clinical Alzheimer's dementia are based on NINCDS/ADRDA criteria.^43^

*Covariables.* We selected as covariates those factors that are: (1) known to be associated with sleep health, cognition, or dementia; (2) not hypothesized to mediate the association of sleep health with cognition or dementia; and (3) reliably measured across both samples as potential confounders.^45^ These selected covariates were: age, subcohort (RS only), race/ethnicity (MAP/MARS only), sex, education, smoking, alcohol, body mass index, and the number of prevalent medical conditions (history of congestive heart failure or heart attack; history of stroke; diabetes; hypertension; and thyroid disease). Self-reported race/ethnicity was only added as a covariate in MAP/MARS as it is not available in RS. A detailed description of the measurement and harmonization of covariates across samples can be found in our previous work.^13,30^ Missing values in covariates occurred in <1% except for body mass index (13.7% in RS; 4.1% in MAP/MARS) and were imputed using the R MissForest package, an iterative imputation method based on a random forest.^46^

### Statistical analysis

*Analytical approach.* We used a coordinated data analysis (also referred to as ‘replication with comparison’^47^) across the two samples. For this, we harmonized sleep health data and covariates as previously described^30^ and applied identical statistical code and models in both samples. We then compared effect sizes across the samples to assess similarities and heterogeneity in findings. With this approach, we focus on the robustness of our findings and possible sources of heterogeneity in the observed effects. While we primarily focused on effect sizes, we considered findings with p < 0.001 to be statistically significant to conservatively account for multiple testing. To explore potential effect modification by sex, we considered p < 0.05 as statistically significant, acknowledging the exploratory nature of these analyses. All analyses were performed using R version 4.3.0 (RS) and 4.4.1 (MAP/MARS).^48^

*Main analyses.* We used Analysis of Covariance (ANCOVA) to determine if standardized cognitive test scores at the initial sleep visit differed across sleep health score levels and sleep heath clusters. To assess the association of multidimensional sleep health with cognitive decline, we regressed repeatedly measured cognition on sleep health, time, sleep health by time interaction, and covariates using linear mixed-effects models with random intercepts. Normality of the cognitive outcomes was evaluated through visual inspection of residual Q-Q plots and the distribution of fitted values. We initially also included natural splines with two degrees of freedom to account for potential non-linear associations with time, which significantly improved model fit in both samples. But, upon visual inspection of the data, we did not observe substantial curvature and decided to prioritize interpretable effect sizes above the added complexity of the splines. Analyses were performed separately with sleep health scores, sleep health clusters, and individual sleep health items as predictors, and were repeated for each cognitive test. Individual sleep health items were tested in separate models. Coefficients with 95% confidence intervals (CI) were reported and, and the yearly change in cognitive performance was estimated with R package emmeans.^49^ Next, we used Cox proportional hazards models to examine the association of sleep health with the risk of incident dementia. Hazard Ratios (HR) and 95%CI were calculated. The proportional hazards assumption was tested by visually inspecting and formally testing the correlation of Schoenfeld residuals with follow-up time and was met for each main exposure in both samples (*p*-values > 0.13). All analyses were adjusted for the previously mentioned covariates.

*Sensitivity analyses.* We performed several additional analyses to assess the robustness of our main findings. First, we assessed potential effect modification by sex by modeling an interaction term and performing stratified analyses when the interaction term was statistically significant. Second, we addressed potential residual confounding by additionally including covariates that can both confound and mediate the association of sleep with cognition and dementia, namely depressive symptoms and use of sedative antidepressants and sedative hypnotics. Third, we repeated analyses specifically for clinical diagnosis of Alzheimer's disease rather than all-cause dementia. Fourth, to account for potential bias by death as a competing risk (i.e., survival bias), we repeated our main analyses with Fine-Gray subdistribution hazard models.^50^ Fifth, to better understand whether differences in the age distribution of RS and MAP/MARS contributed to differences in findings, we decided post hoc to repeat Cox proportional hazard models and Fine-Gray subdistribution hazard models in all participants of RS aged > 75 years at baseline.

## Results

### Sample

We included 7892 participants from the Rotterdam Study and 1442 from MAP/MARS. A detailed description of the study samples is in Table 1. At the initial sleep visit, the mean age (SD) in the RS sample was 69.5 (8.9) years and 58.2% of the sample was female. In MAP/MARS, the mean age was 79.5 (7.9) years and 77.3% of the sample was female. During a mean 10.6 (4.5) years of follow-up in RS, 1148 (14.5%) participants developed dementia. In MAP/MARS, 286 (19.8%) participants developed dementia during a mean 5.3 (2.9) years of follow-up.

**Table 1.** Sample characteristics.

	RS N = 7892	MAP/MARS N = 1442
**Sociodemographic Measures**		
Age	69.5 (8.9)	79.5 (7.9)
Sex - female	58.2% (4592)	77.3% (1114)
Race – White	n.a.	58.9% (850)
African American/Black		40.1% (578)
Other		1.0% (14)
Education – elementary/middle	12.5% (990)	4.0% (57)
High school	43.5% (3433)	44.5% (642)
College	28.1% (2219)	37.7% (543)
Graduate school	15.8% (1250)	13.9% (200)
Marital Status - Married	66.7% (5267)	38.3% (553)
Widowed	19.8% (1562)	36.5% (526)
Other	13.5% (1063)	25.2% (363)
Cohort: - RS-I	42.8% (3375)	
RS-II	30.6% (2412)	
RS-III	26.7% (2105)	
MAP		62.3% (898)
MARS		37.7% (544)
**Physical and Mental Health**		
Smoking status - never	30.1% (2376)	55.1% (794)
former	51.6% (4073)	41.5% (599)
current	18.3% (1443)	3.4% (49)
Alcohol use (score)	3.00 [1.00, 4.00]	0.00 [0.00, 3.00]
Body Mass Index	27.4 [25.2, 29.7]	27.5 [24.5, 31.5]
Number of Comorbidities - 0	44.0% (3473)	21.4% (309)
1	37.1% (2929)	41.4% (597)
2 or more	18.9% (1490)	37.2% (536)
Depressive Symptoms*	4.00 [1.00, 9.00]	1.00 [0.00, 2.00]
Use of sedative antidepressants	1.9% (152)	5.1% (74)
Use of sedative hypnotics	2.2% (173)	3.0% (43)
**Multidimensional Sleep Health**		
Sleep Health Score – 0	46.6% (3677)	25.4% (366)
1	29.4% (2319)	34.7% (501)
2	13.5% (1066)	25.5% (367)
3 or more	10.5% (830)	14.4% (208)
Sleep Health Cluster – Average Sleep	67.8% (5350)	56.2% (811)
Poor sleep	11.7% (927)	8.0% (116)
Inefficient Sleep	20.5% (1615)	35.7% (515)
**Incident Dementia**		
Incident dementia	14.5% (1148)	19.8% (286)
Follow-up time dementia, years	10.6 (4.5)	5.3 (2.9)

Cells show mean (SD), median [Q1, Q3] or % (N). RS: Rotterdam Study; MAP: Memory and Aging Project; MARS: Minority Aging Research Study. * Dutch version of Center for Epidemiologic Studies Depression scale (range 0–60) in RS; Center for Epidemiologic Studies Depression scale (range 0–10) in MAP/MARS.

### Sleep health

**Figure 2.**
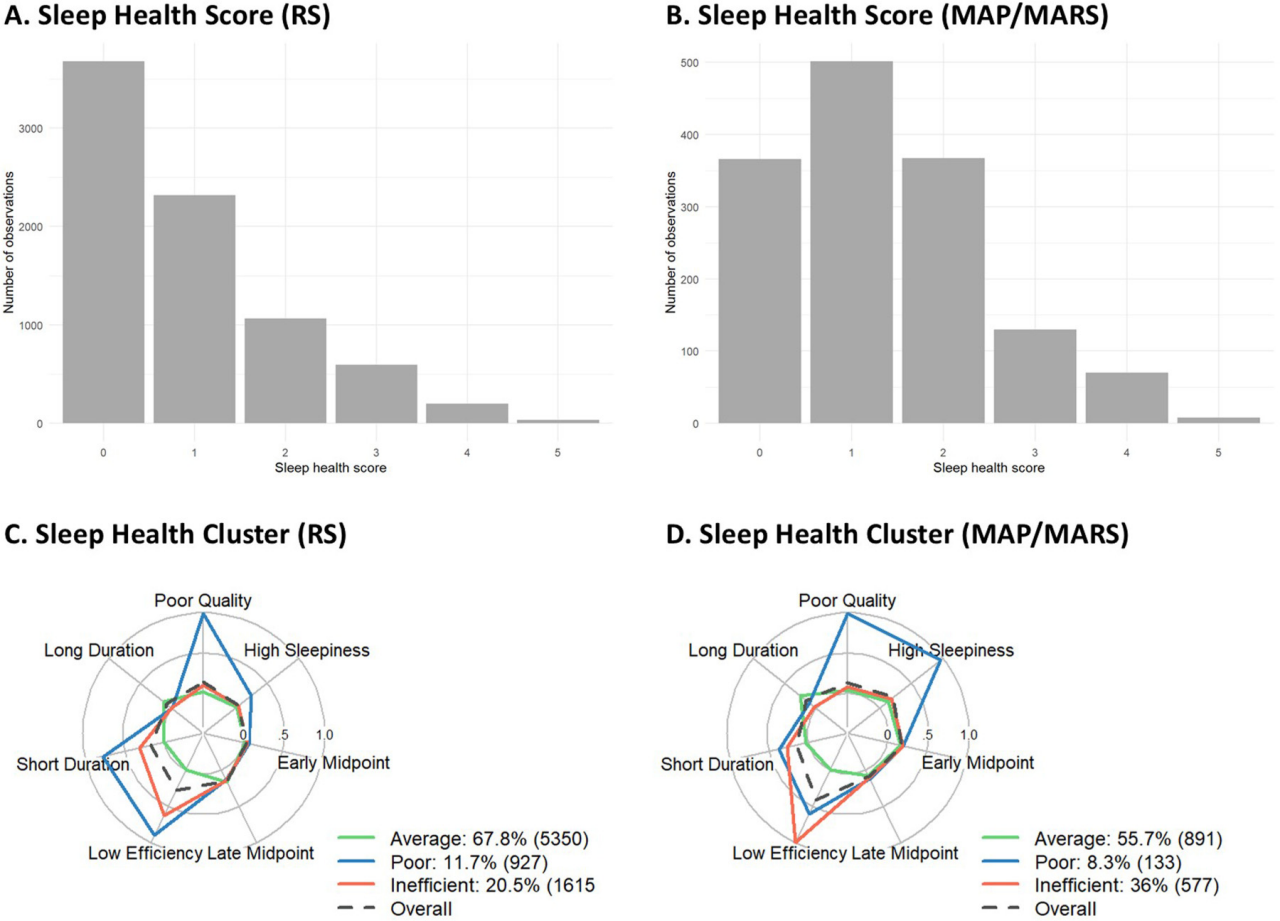
Sleep health clusters and sleep health scores. Distribution of sleep health scores in (A) the Rotterdam Study (RS) and (B) Memory and Aging Project and Minority Aging Research Study (MAP/MARS). Radial plots showing the prevalence of each sleep characteristics by cluster in (C) RS and (D) MAP/MARS, after assigning participants to the cluster for which they had the highest posterior probability. The maximum value 1 indicates that 100% of those assigned to the cluster endorsed that item; whereas the value 0 reflects that no one in that cluster endorsed the item. Average: average sleep health; Poor: poor sleep health; Inefficient: inefficient sleep without complaints; Overall: cohort average.

In both samples, sleep health scores had a right skewed distribution, with more than half of the participants reporting 0–1 poor sleep health items (Table 1; Figure 2). Sleep health clusters were identified with latent class analysis, which was performed separately in RS and MAP/MARS (Figure 2). In both samples, the BIC ‘elbowed’ at the three-cluster solution, suggesting that this fit the data best (Supplemental Table 4). In RS, the 4-cluster solution had similar model fit as the 3-cluster solution. However, upon inspection three of the clusters identified in the 4-cluster solution were similar to those found in the 3-cluster solution; additionally, a small (N = 98, 1.2%) cluster with low stability (Jaccard Index = 0.39) was found, while we defined *a priori* that clusters should be at least 5%. Thus we selected the more robust and stable 3-cluster solution. Upon further inspection, the identified sleep health clusters were similar across the samples and to clusters found in our earlier work.^13^ The three identified clusters were: “Average sleep health” (in RS: 67.8%, n = 5350; in MAP/MARS: 56.2%, n = 811) characterized by good sleep quality, low daytime sleepiness, high efficiency, and average sleep duration; “Poor sleep health” (in RS: 11.7%, n = 927 ; in MAP/MARS: 8.0%, n = 116), characterized by poor sleep quality, high daytime sleepiness, a low sleep efficiency, and short sleep duration; and “Inefficient sleep without complaints” (in RS 20.5: %, n = 1615; in MAP/MARS: 35.7%, n = 515), characterized by good quality, low daytime sleepiness, low efficiency, and short sleep duration. For simplicity, we refer to these as ‘average’, ‘poor’, and ‘inefficient’ sleep clusters. Entropy, Jaccard indices, and average posterior probabilities were all high (> 0.80, except for entropy [0.74] in RS), indicating good separation and stability of clusters (Supplemental Table 5). A network plot of between-cluster distances (Supplemental Figure 2) revealed that clusters in the two samples with the same label (e.g., Inefficient Sleep in RS and MAP/MARS) were consistently more similar to each other than to all other clusters. This indicates that the three cluster types were generalizable across samples.^51^

### Cognitive performance over time

Across the two samples, those who reported one or more poor sleep health items and those with the poor sleep or inefficient sleep cluster generally had poorer cognitive performance across cognitive tests at the initial sleep visit (i.e., cross-sectionally) compared with those with no poor sleep health items or the average sleep cluster (Supplemental Table 6). However, higher sleep health scores and inefficient and poor clusters were consistently not associated with worse cognitive performance over time across RS and MAP/MARS (Table 2). In RS, those who reported 3 or more poor sleep items had a *less* steep decline in performance for the Stroop interference test (change (SD)/year [95%CI]: −0.024 [-0.030; −0.019] compared with those with no poor sleep items (−0.033 [-0.036; −0.031]). A similar pattern was observed for the substitution and category fluency tasks, but not for other tests, in MAP/MARS. However, effect sizes in both samples were generally very small. Given that those with poor sleep health performed worse at the initial visit but had slightly less steep decline over time, cognitive performance over time was similar across levels of sleep health score and clusters.

**Table 2.** Sleep health and cognition.

A. Rotterdam Study					
	Word Learning	Substitution Task	Category Fluency	Stroop processing	Stroop Interference
Total number of observations, *n*	14327	14928	15010	14537	14491
	**Change/year [95% confidence interval]**				
Sleep Health Score					
*0 poor sleep items (Ref.)*	−0.040 [−0.043; −0.037]	−0.051 [−0.053; −0.049]	−0.026 [−0.029; −0.024]	−0.040 [−0.042; −0.038]	−0.033 [−0.036; −0.031]
*1 poor sleep item*	−0.041 [−0.045; −0.037]	−0.049 [−0.052; −0.046]	−0.023 [−0.027; −0.019]	−0.042 [−0.045; −0.039]	−0.032 [−0.035; −0.028]
*2 poor sleep items*	−0.046 [−0.052; −0.039]	−0.049 [−0.054; −0.045]	−0.030 [−0.036; −0.025]	−0.040 [−0.044; −0.036]	−0.041 [−0.046; −0.036]
*3+ poor sleep items*	−0.041 [−0.048; −0.033]	−0.048 [−0.053; −0.043]	−0.025 [−0.031; −0.018]	−0.039 [−0.044; −0.034]	**−0.024 [−0.030; −0.019]**
Sleep health cluster					
*Average sleep (Ref.)*	−0.040 [−0.043; −0.037]	−0.051 [−0.052; −0.049]	−0.025 [−0.028; −0.023]	−0.041 [−0.042; −0.039]	−0.034 [−0.036; −0.031]
*Poor sleep*	−0.039 [−0.046; −0.033]	−0.048 [−0.053; −0.044]	−0.027 [−0.033; −0.021]	−0.039 [−0.044; −0.034]	−0.028 [−0.033; −0.022]
*Inefficient sleep*	−0.046 [−0.051; −0.040]	−0.049 [−0.052; −0.045]	−0.027 [−0.032; −0.022]	−0.041 [−0.045; −0.037]	−0.034 [−0.038; −0.030]
B. MAP/MARS					
	Word Learning	Substitution Task	Category Fluency	Stroop processing	Stroop interference
Total number of observations, *n*	7822	7659	9011	7587	7583
Sleep Health Score					
*0 poor sleep item (Ref.)*	−0.016 [−0.019; −0.014]	−0.032 [−0.034; −0.029]	−0.031 [−0.033; −0.028]	−0.034 [−0.038; −0.030]	−0.028 [−0.031; −0.025]
*1 poor sleep item*	−0.015 [−0.017; −0.012]	−0.028 [−0.030; −0.026]	−0.028 [−0.030; −0.026]	−0.031 [−0.034; −0.027]	−0.028 [−0.030; −0.025]
*2 poor sleep items*	−0.010 [−0.013; −0.007]	−0.026 [−0.029; −0.024]	**−0.023 [−0.026; −0.021]**	−0.025 [−0.030; −0.021]	−0.025 [−0.028; −0.022]
*3+ poor sleep items*	−0.012 [−0.016; −0.008]	**−0.022 [−0.026; −0.019]**	−0.026 [−0.029; −0.022]	−0.024 [−0.030; −0.017]	−0.025 [−0.029; −0.021]
Sleep health cluster					
*Average sleep (Ref.)*	−0.016 [−0.017; −0.014]	−0.029 [−0.031; −0.027]	−0.029 [−0.031; −0.027]	−0.031 [−0.034; −0.029]	−0.027 [−0.029; −0.025]
*Poor sleep*	−0.010 [−0.016; −0.005]	−0.027 [−0.032; −0.022]	−0.025 [−0.030; −0.020]	−0.027 [−0.035; −0.019]	−0.027 [−0.033; −0.021]
*Inefficient sleep*	−0.011 [−0.014; −0.009]	−0.026 [−0.029; −0.024]	−0.025 [−0.027; −0.023]	−0.027 [−0.031; −0.024]	−0.026 [−0.029; −0.023]

Effect sizes reflect the slope of the cognitive trajectory, i.e., the yearly change in cognitive score per year for each category. **Bolded** effect sizes reflect a slope that is significantly different from the reference category (i.e., p < 0.001 & confidence intervals do not overlap). Cognitive test scores were standardized and higher scores reflect better performance. Models were adjusted for age, race (MAP/MARS only), cohort (RS only), sex, education, smoking, alcohol, body mass index, and the number of comorbidities. MAP: Memory and Aging Project; MARS: Minority Aging Research Study.

**Table 3.** Individual domains and change in cognition over time.

A. rotterdam study					
	Word Learning	Substitution Task	Category Fluency	Stroop processing	Stroop Interference
Sleep Health Dimension					
*Good sleep quality (ref.)*	−0.041 [−0.043; −0.039]	−0.050 [−0.052; −0.049]	−0.025 [−0.028; −0.023]	−0.040 [−0.042; −0.039]	−0.034 [−0.036; −0.032]
*Poor sleep quality*	−0.040 [−0.046; −0.034]	−0.049 [−0.053; −0.045]	−0.028 [−0.034; −0.023]	−0.041 [−0.045; −0.037]	−0.029 [−0.034; −0.024]
*Low daytime sleepiness (ref.)*	−0.041 [−0.043; −0.039]	−0.050 [−0.052; −0.049]	−0.026 [−0.028; −0.024]	−0.040 [−0.042; −0.039]	−0.033 [−0.035; −0.031]
*High daytime sleepiness*	−0.037 [−0.048; −0.025]	−0.052 [−0.060; −0.044]	−0.027 [−0.038; −0.016]	−0.041 [−0.049; −0.033]	−0.030 [−0.040; −0.021]
*Early midpoint*	−0.041 [−0.043; −0.038]	−0.050 [−0.052; −0.049]	−0.025 [−0.028; −0.023]	−0.040 [−0.042; −0.039]	−0.033 [−0.035; −0.031]
*Average midpoint (ref.)*	−0.037 [−0.050; −0.025]	−0.045 [−0.053; −0.037]	−0.020 [−0.032; −0.009]	−0.036 [−0.045; −0.028]	−0.031 [−0.041; −0.021]
*Late midpoint*	−0.044 [−0.049; −0.038]	−0.050 [−0.054; −0.047]	−0.029 [−0.034; −0.024]	−0.042 [−0.046; −0.038]	−0.035 [−0.039; −0.030]
*Poor efficiency*	−0.040 [−0.043; −0.038]	−0.051 [−0.052; −0.049]	−0.026 [−0.028; −0.023]	−0.040 [−0.042; −0.039]	−0.033 [−0.035; −0.031]
*Good efficiency (ref.)*	−0.043 [−0.048; −0.039]	−0.048 [−0.051; −0.045]	−0.027 [−0.031; −0.023]	−0.041 [−0.044; −0.038]	−0.032 [−0.035; −0.028]
*Short duration*	−0.040 [−0.043; −0.038]	−0.050 [−0.052; −0.049]	−0.027 [−0.029; −0.024]	−0.041 [−0.043; −0.039]	−0.033 [−0.035; −0.031]
*Average duration (ref.)*	−0.045 [−0.050; −0.039]	−0.048 [−0.052; −0.045]	−0.026 [−0.031; −0.021]	−0.039 [−0.043; −0.035]	−0.032 [−0.037; −0.028]
*Long duration*	−0.038 [−0.046; −0.030]	−0.051 [−0.056; −0.045]	−0.017 [−0.025; −0.010]	−0.038 [−0.043; −0.032]	−0.036 [−0.043; −0.029]
B. MAP/MARS					
	Word Learning	Substitution Task	Category Fluency	Stroop processing	Stroop interference
*Good sleep quality (ref.)*	−0.014 [−0.016; −0.013]	−0.029 [−0.030; −0.027]	−0.028 [−0.029; −0.027]	−0.031 [−0.033; −0.028]	−0.027 [−0.029; −0.025]
*Poor sleep quality*	−0.010 [−0.014; −0.006]	−0.023 [-0.026; −0.019]	−0.022 [−0.025; −0.018]	−0.022 [−0.028; −0.016]	−0.026 [−0.031; −0.022]
*Low daytime sleepiness (ref.)*	−0.013 [−0.015; −0.012]	−0.028 [−0.029; −0.026]	−0.027 [−0.029; −0.026]	−0.030 [−0.032; −0.027]	−0.027 [−0.029; −0.026]
*High daytime sleepiness*	−0.016 [−0.019; −0.012]	−0.030 [−0.033; −0.027]	−0.027 [−0.030; −0.024]	−0.030 [−0.035; −0.025]	−0.025 [−0.029; −0.022]
*Early midpoint*	−0.012 [−0.015; −0.008]	**−0.023 [−0.026; −0.020]**	−0.023 [−0.026; −0.020]	**−0.018 [−0.024; −0.013]**	−0.024 [−0.028; −0.020]
*Average midpoint (ref.)*	−0.015 [−0.016; −0.013]	−0.030 [−0.031; −0.028]	−0.029 [−0.030; −0.027]	−0.032 [−0.035; −0.030]	−0.027 [−0.029; −0.026]
*Late midpoint*	−0.012 [−0.016; −0.007]	−0.023 [−0.028; −0.019]	−0.025 [−0.029; −0.020]	−0.028 [−0.035; −0.021]	−0.−0.028 [−0.032; −0.023]
*Poor efficiency*	−0.015 [−0.017; −0.014]	−0.029 [−0.031; −0.027]	−0.028 [−0.030; −0.027]	−0.031 [−0.033; −0.028]	−0.027 [−0.029; −0.025]
*Good efficiency (ref.)*	−0.011 [−0.013; −0.009]	−0.026 [−0.029; −0.024]	−0.026 [−0.028; −0.023]	−0.028 [−0.031; −0.024]	−0.026 [−0.029; −0.024]
*Short duration*	**−0.007 [−0.011; −0.003]**	**−0.022 [−0.025; −0.018]**	−0.024 [−0.027; −0.020]	−0.024 [−0.029; −0.018]	−0.023 [−0.027; −0.019]
*Average duration (ref.)*	−0.014 [−0.016; −0.012]	−0.029 [−0.030; −0.027]	−0.027 [−0.029; −0.026]	−0.030 [−0.033; −0.028]	−0.027 [−0.029; −0.026]
*Long duration*	**−0.019 [−0.023; −0.015]**	−0.029 [−0.033; −0.026]	−0.033 [−0.036; −0.029]	−0.033 [−0.039; −0.027]	−0.028 [−0.032; −0.024]

Effect sizes reflect the slope of the cognitive trajectory, i.e., the yearly change in cognitive score per year for each category. **Bolded** effect sizes reflect a slope that is significantly different from the reference category, i.e., confidence intervals do not overlap. Cognitive test scores were standardized and higher scores reflect better performance. Models were adjusted for age, race (MAP/MARS only), cohort (RS only), sex, education, smoking, alcohol, body mass index, and the number of comorbidities. MAP: Memory and Aging Project; MARS: Minority Aging Research Study.

Individual sleep health items were generally not associated with cognitive performance over time in RS and MAP/MARS (Table 3). Only in MAP/MARS, those with an early midpoint relative to an average midpoint had a less steep decline in cognitive performance on the substitution task and Stroop processing task (e.g., −0.023 [-0.026; −0.020] versus −0.030 [-0.031; −0.028] for the substitution task), but not for other tasks. In MAP/MARS, short sleep duration compared with average sleep duration was associated with a less steep decline on the word learning and substitution tasks only (e.g., −0.022 [-0.025; −0.018] versus −0.029 [-0.030; −0.027] for the substitution task).

Sex significantly moderated the association of sleep health with performance on the word learning, category fluency, and the Stroop processing test in RS and the word learning test in MAP/MARS (all *p* < 0.001). Estimated yearly changes in cognitive performance on these tests are shown stratified for sex in Supplemental Table 7. For the word learning test in RS and MAP/MARS and category fluency test in RS, trajectories did not differ across levels of sleep or sex. For the Stroop processing test in RS, amongst those with 0 or 1 poor sleep item, males had significantly steeper yearly decline (−0.047 [-0.050; −0.044]) than females (−0.034 [-0.037; −0.032]), but sleep health was associated with slope of cognitive performance in neither males nor females. When main models were additionally adjusted for depressive symptoms and use of sedative medication at the initial visit, effect sizes attenuated and conclusions were consistent (Supplemental Table 8).

### Dementia risk

**Figure 3.**
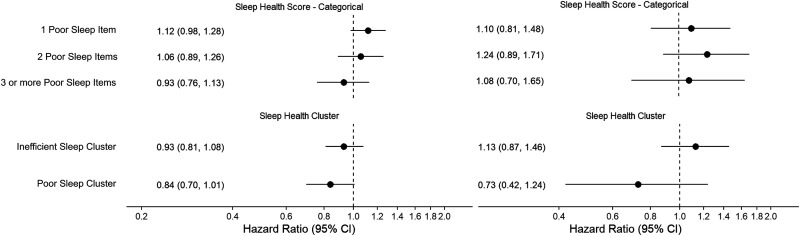
Multidimensional sleep health and risk of dementia. Effect estimates reflect the hazard ratio for dementia compared with the reference category (No poor sleep items/Average sleep cluster). Models were adjusted for age, race (MAP/MARS only), cohort (RS only), sex, education, smoking, alcohol, body mass index, and the number of comorbidities. MAP: Memory and Aging Project; MARS: Minority Aging Research Study.

**Figure 4.**
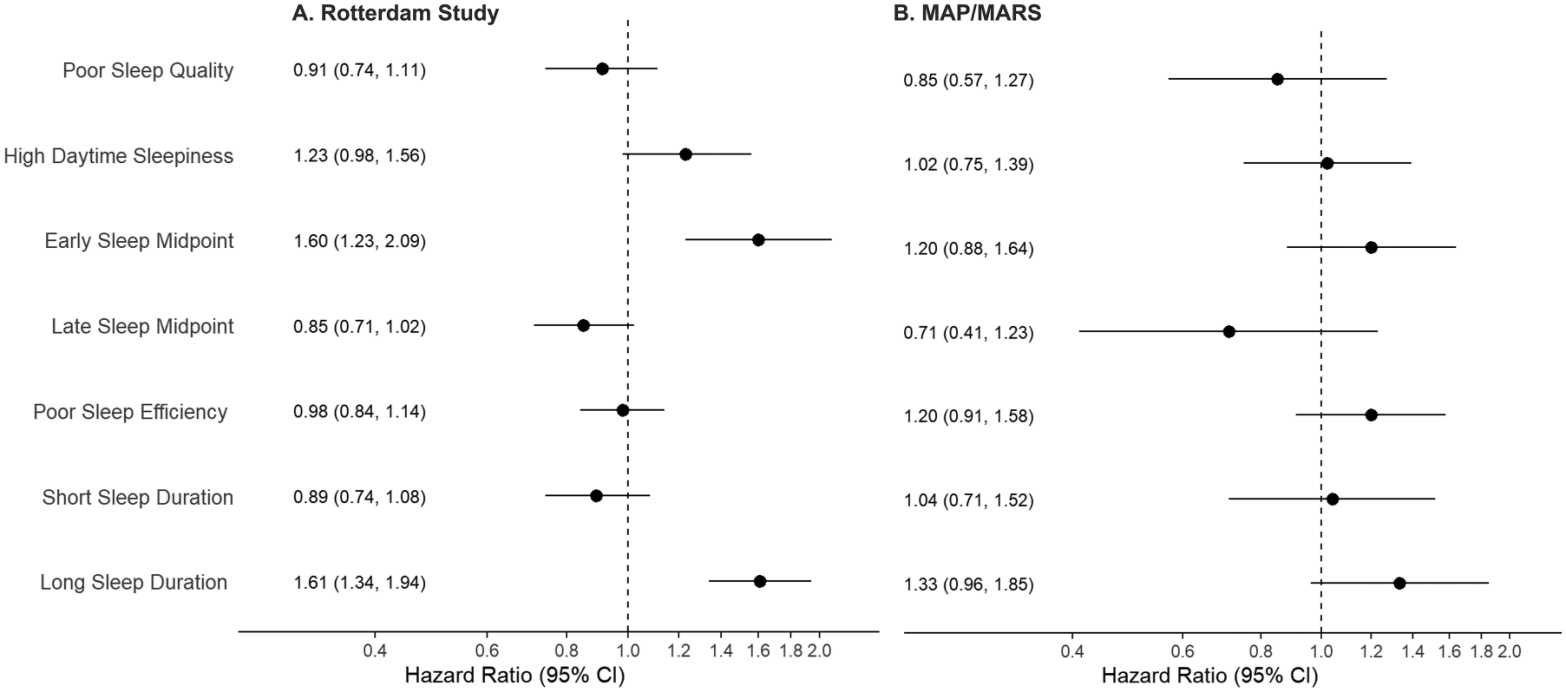
Individual domains and risk of dementia. Effect estimates reflect the hazard ratio for dementia compared with the reference category. Reference categories are: good sleep quality; no daytime sleepiness; average midpoint; good sleep efficiency; and average sleep duration. Models were adjusted for age, race (MAP/MARS only), cohort (RS only), sex, education, smoking, alcohol, body mass index, and the number of comorbidities. MAP: Memory and Aging Project; MARS: Minority Aging Research Study.

Sleep health score was not associated with the risk of dementia in either sample (Hazard Ratio (HR) per each additional poor sleep health measure: 0.99, 95%CI 0.94–1.04 in RS; HR: 1.04, 95%CI 0.93–1.16 in MAP/MARS) (Figure 3, Supplemental Table 9). Similarly, compared with the average cluster, membership of the inefficient sleep cluster or poor sleep cluster was not associated with the risk of dementia in either sample (Inefficient sleep cluster: HR 0.93, 95%CI 0.81–1.08 in RS; HR 1.13, 95%CI 0.87–1.46 in MAP/MARS and Poor sleep cluster: HR 0.84, 95%CI 0.70–1.01 in RS; HR: 0.73, 95%CI 0.42–1.24 in MAP/MARS). Of the individual sleep health items, an early sleep midpoint (HR 1.60; 95%CI 1.23–2.09) and long sleep duration (HR 1.61, 95%CI 1.34–1.94) were associated with a higher risk of dementia in RS (Figure 4A). In MAP/MARS, effect sizes for these items were smaller (HR 1.20, 95%CI 0.88–1.64; HR 1.33, 95%CI 0.96–1.85) and associations were not statistically significant (Figure 4B).

There was a significant interaction of sex with sleep health score in the association with the risk of dementia (*p* = 0.016) in RS, but not in MAP/MARS. Stratified analyses in both samples (Supplemental Table 10) revealed that reporting one poor sleep item relative to no poor sleep items was associated with a higher risk of dementia in females (HR 1.19, 95%CI 1.01–1.41 in RS), but not in males (HR 0.97, 95%CI 0.77–1.22 in RS). Reporting two or more poor sleep health items was not associated with a higher risk of dementia in men or women and there was no interaction of sex with sleep health cluster. When models were additionally adjusted for depressive symptoms and use of sedative medication, effect sizes remained largely similar (Supplemental Figure 3). Conclusions did not change when the outcome was restricted to clinical Alzheimer's disease only (Supplemental Figure 4). Hazard ratios were similar in RS when estimated with Fine-Gray subdistribution hazard models (Supplemental Figure 5). In MAP/MARS, having 3 or more poor sleep items (HR: 1.81, 95%CI 1.23–2.66) or the inefficient sleep cluster (HR: 1.36, 95%CI 1.08–1.73) was associated with a higher risk of dementia when estimated with subdistribution hazard models. When analyses were repeated in a subsample of 2229 participants (mean age: 81.0 [4.5] years; 62.7% female) of RS aged 75 years and older at baseline, the poor sleep (HR 0.79, 95%CI 0.66–0.96) and inefficient sleep cluster (HR 0.77, 95%CI 0.61–0.98) were associated with a lower risk of dementia, although effects did not reach our set threshold for significance after multiple comparison adjustment (*p* = 0.013) (Supplemental Table 1^1^). Sleep health score was not associated with risk of dementia and effect sizes for Fine-Gray subdistribution models were similar.

## Discussion

In two population-based samples of older adults, multidimensional sleep health—indicated by either composite self-reported sleep health scores or sleep health clusters—was not consistently associated with cognitive decline or dementia. Although a poorer sleep health score was associated with worse cognitive performance at the time of the sleep measurement, cognitive trajectories over time were similar across levels of sleep health. Sleep health was also not associated with time to dementia in our primary models.

Our findings are inconsistent with two prior studies of self-reported sleep health and dementia or cognitive decline. Specifically, in the Osteoporotic Fractures in Men (MrOS) study^27^ and in the UK biobank,^29^ a worse self-reported sleep health score was associated with greater cognitive decline and a higher risk of dementia, respectively. However, our findings are consistent with those from a sample of middle-aged and older adults, which did not observe associations between self-reported sleep health and cognitive functioning after 9 years.^28^ These discrepant findings across samples likely results from the use of different sleep health domains and specific items across studies, which hinders comparisons of the findings and warrants future efforts to standardize sleep health measurement.^30^ For example, the analysis in the UK biobank^29^ included snoring and napping in their sleep health score, but not sleep efficiency. Moreover, demographic differences may explain observed discrepancies with previous work, as the MrOS cohort consists of older men and the UK biobank cohort is slightly younger (average age: 60.5 years) than RS and MAP/MARS. Thus, we suspect that the continued inconsistencies in the literature stem both from the heterogeneity in methods for measuring sleep health as well as nuances in the associations between sleep health, cognition and dementia.

We advanced prior work by analyzing sleep health clusters, which reflect within-person patterns of sleep health characteristics. We identified three clusters—average sleep, inefficient sleep, and poor sleep—that were previously found to generalize across six independent samples.^13^ Huang et al.^29^ similarly performed LCA in the much larger sample of the UK Biobank but without validation in an independent sample. Using a somewhat different set of self-reported sleep indicators than in our report, they identified a cluster of relatively healthy sleep (67.0%), comparable in size and characteristics to the average sleep cluster in this work (56.2%-67.8%), as well as two medium-sized (∼10–12%) and three smaller (∼1–5%) sleep health clusters with more extreme sleep health patterns, which were associated with a slightly higher risk of dementia. Associations with cognitive decline were not tested. Overall, the effect sizes of the medium-sized clusters with dementia were modest (HR: 1.08–1.19), which together with our work seems to suggest that these more generalizable clusters may not pose clinically meaningful risk differences. Huang et al.'s ‘severely disturbed sleep’ cluster—characterized by napping, long sleep duration, daytime sleepiness, insomnia symptoms, and non-restorative sleep—showed a stronger association with dementia. However, this cluster reflected a rare (1.0%) and more severe phenotype, which might have not been picked up in our independent samples of smaller size, and which may be less likely to generalize to other samples as well. Therefore, we tentatively conclude that common and generalizable composite measures of self-report sleep health such as those we present here may not help to identify individuals with an elevated risk of cognitive decline or dementia. Clusters representing extreme ends of sleep health spectrum may be informative, but such clusters must be replicated.

Placing our current findings in the context of the broader literature on sleep, cognitive aging, and dementia, prior reported associations may have been driven by severe sleep disturbances or sleep disorders, rather than by sleep health more generally. Indeed, several meta-analyses demonstrate that those with sleep disturbances and sleep disorders have a higher risk of cognitive decline, dementia, and Alzheimer's disease.^16–18^ In line with Buysse et al.,^1^ we defined multidimensional sleep health as the within-person pattern of satisfaction, alertness, timing, efficiency and duration, independent of whether a sleep disorder was present. Previous studies demonstrated that sleep health scores and clusters are associated with other adverse health outcomes including mortality, cardiovascular health, and depressive symptoms adults,^2–4^^,12,13,52,53^ above and beyond individual sleep items. The observation that worse sleep health scores predict some health outcomes but not others underscores that the definition of ‘poor’ versus ‘good’ sleep health may be outcome specific. Thus, for example, sleep health recommendations for depression might differ from recommendations for dementia.

The individual sleep health items long sleep duration and early midpoint were associated with a higher risk of dementia in both samples, in line with previous literature,^16,54–59^ although effect sizes were smaller and not significant in MAP/MARS. Long sleep duration and earlier sleep timing may reflect increased sleep drive, i.e., hypersomnia or a circadian shift, which could be an early marker of underlying neurodegenerative processes.^15,56,60,61^ Indeed, early sleep timing and a shift to a longer sleep duration correlate with lower hippocampal volumes and markers of cerebrovascular pathology.^62,63^ Other items were not consistently associated with cognitive outcomes, although based on previous evidence one might expect short sleep duration, inefficient sleep, or poor quality to predict dementia.^16–18^ Again, findings in the literature might be driven by more severe sleep disturbances (e.g., a sleep duration < 5 h^16,54^). Our findings suggest that specific poor sleep health items, such as self-reported long sleep duration and early timing, are potentially more informative than generalizable composite sleep health measures to signal heightened cognitive risk in older adults. In composite measures of sleep health the predictive effects of these individual sleep health items might be washed out by the “noise” of other items, resulting in a weak or counterintuitive association of the composite measure.^11^ This could be partially overcome in future work with outcome-specific weights for sleep health items or machine learning models that account for complex interactions, although these steps can limit generalizability and applicability of measures.^4,53,64^ For example, other groups have successfully identified novel dementia risk factors such as heart rate using machine learning models.^65,66^

Future studies could extend our findings by incorporating objective measures of sleep. We based our sleep health measure on self-reported sleep due to its accessibility in clinical practice, but several key aspects of sleep, such as sleep fragmentation, disrupted 24-h activity rhythms, and micro- and macro sleep architecture, cannot be reliably captured through self-report. These dimensions have been suggested to predict cognitive decline and dementia.^14,67–70^ In RS, actigraphy-based measures of poor sleep, but not subjective sleep quality, were previously associated with dementia,^59,71^ although a meta-analysis did not observe differences in the association with dementia between self-reported and objective measures of sleep.^18^ Several studies used person-centered methods to identify clusters of actigraphy-based sleep and circadian rhythm parameters (e.g.,^72,73^). In the MrOS cohort, a cluster of fragmented, poor sleepers was associated with an increased risk of dementia compared with those with average sleep.^74^ However, findings have not yet been replicated in independent samples.

Furthermore, our sensitivity analyses highlight several nuances to our findings. First, sex might modify the association of sleep health with the risk of dementia, although findings were only statistically significant in RS. Reporting one poor sleep health item was associated with a higher risk of dementia only in women. This corresponds with evidence suggesting that women may be more vulnerable to the potential effects of poor sleep on brain health, possibly as the result of hormonal changes in menopause which affect both sleep and brain health^75,76^ and underscoring the importance of investigating sex-specific associations of sleep with brain health.^77^ Moreover, when estimated with Fine-Gray subdistribution hazard models to account for competing risk of death, poor sleep health was associated with a higher risk of dementia in MAP/MARS. This was observed in neither the main nor the older subsample of RS. The discrepancy between the two samples may result from the different methods used to monitor for dementia cases and warrants replication in independent cohorts.

An important strength of this study is the coordinated analysis across two harmonized samples that are analyzed independently. This approach improves the rigor and validity of our findings while making optimal use of the strengths of the individual samples. Accordingly, we focus on interpreting findings that are most consistent across samples. A limitation is that discrepant findings can be difficult to interpret, as they can reflect true differences, imperfect harmonization, or chance.^47^ Several other limitations of our work should be mentioned. First, comparison of included and excluded participants indicated potential selection bias in the linear mixed models in RS, which may also limit generalizability of the results. Sensitivity analyses indicate that selection bias, and specifically survival bias, may have affected estimated effect sizes and may have led to over- or underestimating the true effects of the associations, warranting further replication in independent samples. Second, even though our aim was identification rather than causal inference, we emphasize that associations of self-report sleep health with cognitive performance and dementia in older adults are likely affected by reverse causation, as illustrated by the baseline differences in cognitive performance across sleep health groups. Similarly, although we adjusted for a wide range of potential confounders, residual confounding may bias these associations. Last, effect sizes shifted substantially when models were additionally adjusted for depressive symptoms at the initial visit. Depressive symptoms can confound, mediate, and moderate the association of sleep with cognitive outcomes.^17,78^ Given that depressive symptoms are highly prevalent amongst older adults and poor sleep may be a modifiable risk factor for depressive symptoms,^79,80^ these temporal pathways should be disentangled in dedicated future studies with repeatedly measured dimensions of sleep health, depressive symptoms, and cognitive status.

### Conclusion

In this coordinated analysis of two large population-based samples, composite measures of multidimensional sleep health were not associated with cognitive decline or dementia. Based on the findings in these samples, composite measures of self-reported multidimensional sleep health do not appear to be informative screening measures for those with an elevated risk of cognitive decline and dementia. Future studies should use more rigorous and standardized definitions of sleep health to enhance reproducibility and could improve predictive power by focusing on more severe sleep health phenotypes, using individual sleep health dimensions, more complex modeling techniques or including objective measures of sleep.

## Supplemental Material

sj-docx-1-alz-10.1177_13872877261422263 - Supplemental material for Multidimensional self-reported sleep health, cognitive decline, and risk of all-cause dementia: A population-based multi-cohort study Supplemental material, sj-docx-1-alz-10.1177_13872877261422263 for Multidimensional self-reported sleep health, cognitive decline, and risk of all-cause dementia: A population-based multi-cohort study by Sanne J. W. Hoepel, Nina Oryshkewych, Lisa L. Barnes, Meryl Butters, Daniel Buysse, M. Kamran Ikram, Andrew Lim, Frank J. Wolters, Lan Yu, Meredith L. Wallace and Annemarie I. Luik in Journal of Alzheimer's Disease
