# Supplementary material for: Multidimensional self-reported sleep health, cognitive decline, and risk of all-cause dementia: A population-based multi-cohort study
Source: J Alzheimers Dis. 2026 Mar 16;110(3):1127–42. doi: 10.1177/13872877261422263 (PMC13022024; doi:10.1177/13872877261422263)
Supplement: sj-docx-1-alz-10.1177_13872877261422263 - Supplemental material for Multidimensional self-reported sleep health, cognitive decline, and risk of all-cause dementia: A population-based multi-cohort study [file sj-docx-1-alz-10.1177_13872877261422263.docx]

**Supplemental Material**

**Multidimensional self-reported sleep health, cognitive decline, and risk of all-cause dementia: A population-based multi-cohort study**

##

## **Supplemental Figure 1.** Flowchart of the study samples.

Did not participate in cognitive testing at baseline

Rotterdam Study**: N = 912**

MAP/MARS: **N = 0**

Prevalent dementia at baseline

Rotterdam Study**: N = 77**

MAP/MARS: **N = 68**

Incomplete sleep health or dementia data at baseline

Rotterdam Study**: N = 396**

MAP/MARS: **N = 165**

**Sample for repeated cognition analysis**

Rotterdam Study**: N = 6980 (83.4%)**

MAP/MARS: **N = 1442 (85.7%)**

**Main study sample:** Complete sleep and dementia data and free of dementia

Rotterdam Study**: N = 7892 (94.3%)**

MAP/MARS: **N = 1442 (85.7%)**

Participated in the baseline interview

Rotterdam Study**: N = 8365 (100%)**

MAP/MARS: **N = 1683 (100%)**

Complete sleep and dementia data

Rotterdam Study**: N = 7969 (95.3%)**

MAP/MARS: **N = 1518 (90.2%)**

## **Supplemental Table 1.** Baseline differences between participants with and without baseline cognitive test data in the Rotterdam Study.

|  | **Cognitive tests performed**  N = 6980 | **No cognitive tests performed at baseline**  N = 912 |  |
| --- | --- | --- | --- |
| **Sociodemographic Measures** |  |  | *p*-value for difference |
| Age | 68.6 (8.47) | 76.1 (9.68) | <0.001 |
| Sex - female | 57.4% (4005) | 64.4% (587) | <0.001 |
| Education – elementary/middle | 11.4% (798) | 21.1% (192) | <0.001 |
| - High school | 43.7% (3051) | 41.9% (382) |  |
| - College | 28.4% (1982) | 26.0% (237) |  |
| - Graduate school | 16.5% (1149) | 11.1% (101) |  |
| Marital Status - Married | 68.9% (4809) | 50.2% (458) | <0.001 |
| Widowed | 17.6% (1231) | 36.3% (331) |  |
| Other | 13.5% (940) | 13.5% (123) |  |
| Cohort: - RS-I | 40.6% (2835) | 59.2% (540) | <0.001 |
| RS-II | 30.5% (2132) | 30.7% (280) |  |
| RS-III | 28.8% (2013) | 10.1% (92) |  |
| **Physical and Mental Health** |  |  |  |
| Smoking status - never | 29.5% (2056) | 35.1% (320) | <0.001 |
| - former | 52.6% (3669) | 44.3% (404) |  |
| - current | 18.0% (1255) | 20.6% (188) |  |
| Alcohol use (score) | 2.00 [0, 4.00] | 3.00 [1.00, 4.00] | <0.001 |
| Body Mass Index | 27.7 (4.21) | 27.9 (1.89) | 0.17 |
| Number of Comorbidities - 0 | 45.7% (3188) | 31.3% (285) | <0.001 |
| - 1 | 37.0% (2583) | 37.9% (346) |  |
| - 2 or more | 17.3% (1209) | 30.8% (281) |  |
| Depressive Symptoms | 5.92 (7.43) | 9.86 (9.43) | <0.001 |
| Use of sedative antidepressants | 1.7% (122) | 3.3% (30) | 0.002 |
| Use of sedative hypnotics | 2.1% (148) | 2.7% (25) | 0.28 |
| **Multidimensional Sleep Health** |  |  |  |
| Sleep Health Score – 0 | 47.3% (3302) | 41.1% (375) | <0.001 |
| - 1 | 29.4% (2049) | 29.6% (270) |  |
| - 2 | 13.2% (918) | 16.2% (148) |  |
| - 3 or more | 10.2% (711) | 13.0% (119) |  |
| Sleep Health Cluster – Average Sleep | 68.7% (4797) | 60.6% (553) | <0.001 |
| Poor sleep | 11.4% (798) | 14.1% (129) |  |
| Inefficient Sleep | 19.8% (1385) | 25.2% (230) |  |

Cells show mean (SD), median [Q1, Q3] or % (N).

## **Supplemental Table 2.** Baseline difference between participants with and without complete data on sleep health and dementia at baseline in MAP/MARS.

|  | **Included in main analysis**  N = 1442 | **Excluded due to incomplete sleep health or dementia data at baseline**  N = 159 |  |
| --- | --- | --- | --- |
| **Sociodemographic Measures** |  |  | *p*-value for difference |
| Age | 79.5 (7.86) | 79.0 (7.81) | 0.456 |
| Sex - female | 77.3% (1114) | 77.4% (123) | 0.99 |
| Education – elementary/middle | 4.0% (57) | 5.0% (8) | 0.439 |
| - High school | 44.5% (642) | 46.5% (74) |  |
| - College | 37.7% (543) | 39.0% (62) |  |
| - Graduate school | 13.9% (200) | 9.4% (15) |  |
| Marital Status - Married | 38.3% (553) | 39.0% (62) | 0.665 |
| Widowed | 36.5% (526) | 39.6% (63) |  |
| Other | 25.4 % (363) | 21.4% (34) |  |
| Cohort: - MAP | 62.3% (898) | 56.0% (89) | 0.143 |
| MARS | 37.7% (544) | 44.0% (70) |  |
|  |  |  |  |
| **Physical and Mental Health** |  |  |  |
| Smoking status - never | 55.1% (794) | 51.6% (82) | 0.701 |
| - former | 41.5% (599) | 44.7% (71) |  |
| - current | 3.4% (49) | 3.8% (6) |  |
| Alcohol use (score) | 1.37 (1.69) | 1.42 (1.66) | 0.688 |
| Body Mass Index | 28.4 (6.01) | 29.1 (6.33) | 0.231 |
| Number of Comorbidities - 0 | 21.4% (309) | 20.1% (32) | 0.746 |
| - 1 | 41.4% (597) | 39.6% (63) |  |
| - 2 or more | 37.2% (536) | 40.3% (64) |  |
| Depressive Symptoms | 8.0% (115) | 8.8% (14) | 0.833 |
| Use of sedative antidepressants | 5.1% (74) | 8.2% (13) | 0.155 |
| Use of sedative hypnotics | 3.0% (43) | 1.3% (2) | 0.319 |

Cells show mean (SD), median [Q1, Q3] or % (N).

## **Supplemental Table 3.** Harmonization of cognitive tests.

| **Task** | **Rotterdam Study** | **MAP/MARS** |
| --- | --- | --- |
| Word Learning | **15 Word Learning Test – delayed recall** | **Word list** |
|  | Participant is presented with 15 words | The participant is asked to read a list of 10 words one at a time. They are presented with three trials with the words in different order for each trial. A few minutes later the participant is asked to recall as many words as possible. |
|  | The total score (0-15) is the number of correct words recalled, with higher scores indicating better performance. | The total score (0-10) is the number of correct words recalled, with higher scores indicating better performance. |
| Substitution | **Letter digit Substitution Task** | **Symbols digits Modality Test** |
|  | The participant is presented with a letter and has to write down a digit according to a presented key as quickly as possible. | The participant is presented with a series of abstract symbols and is asked to identify and call out the corresponding number, according to a presented key as quickly as possible. |
|  | The total score is the number of correctly identified letters within a 60-second time limit, with higher scores indicating better performance | The total score is the number of correctly identified symbols, with higher scores indicating better performance. |
| Verbal Fluency | **Category Fluency Test** | **Category Fluency test** |
|  | Participants are asked to generate exemplars from a category (animals) within a 60-second time limit. | Participants are asked to generate exemplars from each of two categories (animals, fruits and vegetables) within a 60-second time limit. |
|  | The variable is the number of unique animals named, with higher scores indicating better performance. | The variable is the number of unique animals named, with higher scores indicating better performance. |
| Stroop Processing | **Stroop Task (color & word naming)** | **Stroop Task (color & word naming)** |
|  | Consists of 3 trials in which the participant has to name 40 items shown on a card. In trial 1, the card contains color-names printed in black and participants are asked to read aloud the printed word. In trial 2, the card contains colored blocks and participants are asked to name the printed color.  The time needed per trial is noted, with a maximum of 300 seconds. | Participants are asked to read aloud a list of words as quickly as they can, within a 30-second time limit. |
|  | The total score is the time needed per trial, averaged over trial 1 and trial 2, with higher sores indicate worse performance. | The total score is the number of words read correctly, with higher scores indicating better performance. |
| Stroop Interference | **Stroop Task (interference score)** | **Stroop Task (interference score)** |
|  | In trial 3, the card contains color-names printed in a different color than the color-name and participants are asked to name the color of the ink.  The time needed per trial is noted, with a maximum of 300 seconds. | Participants are asked to name aloud the color of ink that each word in the list is printed in as quickly as they can within a 30-second time limit. |
|  | Calculated as the difference between the timed needed to finish trial 3 and the average time needed to finish trial 1 and 2, with higher scores indicate worse performance (i.e., more interference). | The score is the total number of colors correctly named, with higher scores indicating better performance. |

## **Supplemental Table 4.** Clustering analysis model fit statistics.

| **Cohort** | **No. of Clusters** | **AIC** | **BIC** |
| --- | --- | --- | --- |
| RS | 1 | 39647.48 | 39696.30 |
|  | 2 | 35646.83 | 35751.43 |
|  | 3 | 35470.97 | 35631.36 |
|  | 4 | 35393.23 | 35609.41 |
|  | 5 | 35333.23 | 35605.21 |
|  | 6 | 35313.71 | 35641.47 |
| MAP/MARS | 1 | 10244.51 | 10282.16 |
|  | 2 | 9856.89 | 9937.56 |
|  | 3 | 9740.70 | 9864.40 |
|  | 4 | 9723.77 | 9890.49 |
|  | 5 | 9724.71 | 9934.46 |
|  | 6 | 9728.18 | 9980.96 |

## **Supplemental Table 5.** Sleep health clusters.

| 1. **Rotterdam Study** | **Average sleep** | **Poor sleep** | | **Inefficient sleep** | |  |
| --- | --- | --- | --- | --- | --- | --- |
| % (N) | 67.8% (5350) | 11.7% (927) | | 20.5% (1615) | |  |
| Stability (Jaccard Index) | 0.96 | 0.83 | | 0.83 | |  |
| Average posterior probability | 0.89 | 0.81 | | 0.98 | |  |
| Entropy | 0.74 | | | |  |  |
|  | **Response N (%)** | | **Overall** | | | |
|  |  |  | |  | |  |
| Poor sleep quality | 2.7% (144) | 100% (927) | | 0.5% (8) | | 13.7% (1079) |
| High daytime sleepiness | 2.1% (111) | 23.5% (218) | | 4.9% (79) | | 5.2% (408) |
| Early sleep midpoint | 2.9% (153) | 6.9% (64) | | 6.3% (101) | | 4.0% (318) |
| Late sleep midpoint | 16.1% (860) | 14.0% (130) | | 14.2% (229) | | 15.4% (1219) |
| Low sleep efficiency | 0% (0) | 89.4% (829) | | 84.4% (1363) | | 27.8% (2192) |
| Short sleep duration | 0% (0) | 72.0% (667) | | 41.9% (676) | | 17.0% (1343) |
| Long sleep duration | 12.0% (641) | 0.2% (2) | | 0.6% (10) | | 8.3% (653) |
| Sleep health score, *median [Q1, Q3]* | 0 [0, 1.00] | 3.00 [3.00, 3.00] | | 1.00 [1.00, 2.00] | | 1.00 [0, 1.00] |

| 1. **MAP/MARS** | **Average sleep** | **Poor sleep** | **Inefficient sleep** | |  |
| --- | --- | --- | --- | --- | --- |
| % (N) | 56.2% (811) | 8.0% (116) | 35.7% (515) | |  |
| Stability (Jaccard Index) | 0.98 | 0.89 | 0.95 | |  |
| Average posterior probability | 0.99 | 0.86 | 0.94 | |  |
| Entropy | 0.89 | | |  |  |
|  | **Response N (%)** | | **Overall** | | |
|  |  |  |  | |  |
| Poor sleep quality | 0.0% (0) | 90.1% (115) | 7.6% (39) | | 13.1% (189) |
| High daytime sleepiness | 13.4% (109) | 95.7% (111) | 19.6% (101) | | 22.3% (321) |
| Early sleep midpoint | 18.0% (146) | 19.8% (23) | 19.8% (102) | | 18.8% (271) |
| Late sleep midpoint | 7.5% (61) | 12.1% (14) | 10.3% (53) | | 8.9% (128) |
| Low sleep efficiency | 0% (0) | 61.2% (71) | 100% (515) | | 40.6% (586) |
| Short sleep duration | 3.2% (26) | 39.7% (46) | 28.3% (146) | | 15.1% (218) |
| Long sleep duration | 25.2% (204) | 9.5% (11) | 3.3% (17) | | 16.1% (232) |
| Sleep health score, *median [Q1, Q3]* | 1.00 [0, 1.00] | 3.00 [3.00, 4.00] | 2.00 [1.00, 2.00] | | 1.00 [0, 2.00] |

MAP: Memory and Aging Project; MARS: Minority Aging Research Study. Jaccard Index: Quantifies the stability of each cluster through iterative bootstrapping, re-clustering, and comparison to the original cluster assignment, values closer to 1 reflect higher stability. Average Posterior Probability: The average of the posterior probability for all participants within a given cluster. Clusters with values closer to 1 are more distinct from the others, while clusters with values closer to 0 are more highly overlapping clusters. Entropy: Summarizes the overall precision of assigning cluster membership in the sample, derived based on the estimated posterior probabilities. Relative entropy equals 0 when the classification is no better than random guessing (i.e., maximum uncertainty, with potentially problematic over-extraction of clusters) and 1 when the classification is perfect (i.e., maximum certainty, with highly-differentiated clusters).

## **Supplemental Figure 2.** Network plot of cluster distances.


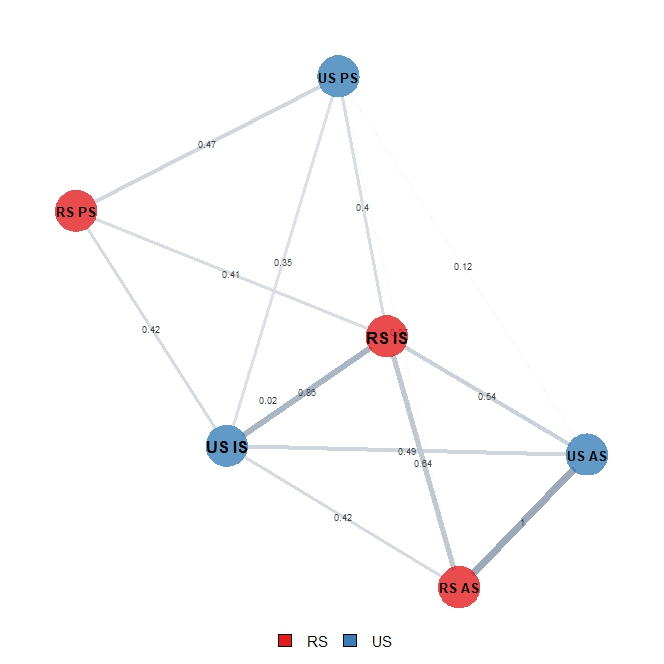


Rescaled & reversed Wasserstein distances, reflecting the similarity of clusters. Scaled to 1 = most similar clusters present. US = MAP/MARS sample; RS = Rotterdam Study. Clusters with the same label are more similar to each other across sample than to other clusters from the same sample.

##

## **Supplemental Table 6.** Cognitive performance at initial sleep visit.

| 1. **Rotterdam Study** |  | | | | | | |
| --- | --- | --- | --- | --- | --- | --- | --- |
|  | **Word Learning** | **Substitution Task** | **Category Fluency** | **Stroop processing** | **Stroop Interference** |  |  |
| Sleep Health Score |  |  |  |  |  |  |  |
| *0 poor sleep items (Ref.)* | 0.154 (1.04) | 0.247 (1.01) | 0.204 (1.04) | 0.176 (0.94) | 0.216 (0.89) |  |  |
| *1 poor sleep item* | 0.065 (1.02) | 0.119 (1.02) | 0.087 (1.06) | 0.112 (0.96) | 0.080 (0.97) |  |  |
| *2 poor sleep items* | 0.142 (1.03) | 0.068 (1.06) | 0.108 (1.10) | 0.083 (1.03) | 0.072 (0.99) |  |  |
| *3+ poor sleep items* | 0.152 (1.06) | 0.057 (1.04) | 0.067 (1.07) | 0.112 (1.00) | 0.052 (0.95) |  |  |
|  | *p* = 0.024 | ***p* < 0.001** | ***p* < 0.001** | *p* = 0.024 | ***p* < 0.001** |  |  |
| Sleep health cluster |  |  |  |  |  |  |  |
| *Average sleep (Ref.)* | 0.126 (1.04) | .208 (1.02) | 0.166 (1.06) | 0.154 (0.96) | 0.174 (0.92) |  |  |
| *Poor sleep* | 0.164 (1.06) | .098 (1.05) | 0.097 (1.09) | 0.128 (0.94) | 0.113 (0.88) |  |  |
| *Inefficient sleep* | 0.106 (1.01) | .061 (1.03) | 0.089 (1.04) | 0.092 (1.00) | 0.042 (1.01) |  |  |
|  | *p* = 0.48 | ***p* < 0.001** | *p* = 0.028 | *p* = 0.12 | ***p* < 0.001** |  |  |
| 1. **MAP/MARS** |  | | | | | |  |
|  | **Word Learning** | **Substitution Task** | **Category Fluency** | **Stroop processing** | **Stroop interference** |  |  |
| Sleep Health Score |  |  |  |  |  |  |  |
| *0 poor sleep items (Ref.)* | 0.017 (0.249) | 0.058 (0.263) | 0.058 (0.258) | 0.072 (0.394) | 0.0340 (0.263) |  |  |
| *1 poor sleep item* | 0.003 (0.247) | -0.003 (0.288) | 0.001 (0.276) | 0.010 (0.414) | 0.007 (0.279) |  |  |
| *2 poor sleep items* | -0.013 (0.243) | -0.011 (0.281) | -0.029 (0.275) | -0.039 (0.411) | -0.012 (0.283) |  |  |
| *3+ poor sleep items* | -0.016 (0.236) | -0.077 (0.294) | -0.054 (0.254) | -0.085 (0.387) | -0.058 (0.281) |  |  |
|  | *p* = 0.286 | ***p* < 0.001** | ***p* < 0.001** | ***p* < 0.001** | *p* = 0.002 |  |  |
| Sleep health cluster |  |  |  |  |  |  |  |
| *Average sleep (Ref.)* | 0.011 (0.248) | 0.021 (0.280) | 0.027 (0.269) | 0.040 (0.411) | 0.021 (0.270) |  |  |
| *Poor sleep* | -0.018 (0.238) | -0.073 (0.295) | -0.053 (0.271) | -0.044 (0.375) | -0.013 (0.270) |  |  |
| *Inefficient sleep* | -0.013 (0.242) | -0.017 (0.282) | -0.030 (0.268) | -0.053 (0.403) | -0.031 (0.288) |  |  |
|  | *p* = 0.17 | ***p* < 0.001** | ***p* < 0.001** | ***p* < 0.001** | *p* = 0.004 |  |  |

Cognitive test scores were standardized and are presented across levels of sleep health score and cluster. Higher scores reflect better performance. ANCOVA was used to determine if test scores varied across different levels of sleep health scores and clusters, reflected here with *p*-values. MAP: Memory and Aging Project; MARS: Minority Aging Research Study.

## **Supplemental Table 7.** Sleep health and cognition – Stratified by sex.

|  |  |  | |  | |  | | | |  | | |  |  |
| --- | --- | --- | --- | --- | --- | --- | --- | --- | --- | --- | --- | --- | --- | --- |
| **A. Rotterdam Study** | |  | |  | |  | | | |  | | |  |  |
|  | **Word Learning** | | | **Category Fluency** | | | | | **Stroop processing** | | | | |  |
|  | Change/year [95% confidence interval] | | | Change/year [95% confidence interval] | | | | | Change/year [95% confidence interval] | | | | |  |
|  | **Males** | **Males** | | **Males** | **Females** | | | **Males** | | | | **Females** | | |
| Sleep Health Score |  |  | |  |  | | |  | | | |  | | |
| *0 poor sleep items (Ref.)* | -0.040 [-0.044; -0.035] | -0.041 [-0.045; -0.037] | | -0.028 [-0.032; -0.024] | -0.025 [-0.029; -0.021] | | | **-0.047 [-0.050; -0.044]^a^** | | | | **-0.034 [-0.037; -0.032]^a^** | | |
| *1 poor sleep item* | -0.044 [-0.050; -0.038] | -0.039 [-0.044; -0.033] | | -0.023 [-0.029; -0.017] | -0.024 [-0.029; -0.019] | | | **-0.050 [-0.054; -0.045]^a^** | | | | **-0.036 [-0.039; -0.032]^a^** | | |
| *2 poor sleep items* | -0.052 [-0.063; -0.041] | -0.043 [-0.051; -0.035] | | -0.029 [-0.040; -0.019] | -0.031 [-0.038; -0.024] | | | -0.045 [-0.052; -0.037] | | | | -0.038 [-0.043; -0.033] | | |
| *3+ poor sleep items* | -0.044 [-0.059; -0.030] | -0.040 [-0.048; -0.032] | | -0.017 [-0.032; -0.003] | -0.027 [-0.035; -0.020] | | | -0.042 [-0.052; -0.031] | | | | -0.038 [-0.044; -0.033] | | |
| Sleep health cluster |  |  | |  |  | | |  | | | |  | | |
| *Average sleep (Ref.)* | -0.041 [-0.045; -0.038] | -0.039 [-0.043; -0.036] | | -0.027 [-0.030; -0.023] | -0.025 [-0.028; -0.021] | | | -0.048 [-0.051; -0.045] | | | | -0.034 [-0.036; -0.032] | | |
| *Poor sleep* | -0.042 [-0.057; -0.027] | -0.040 [-0.047; -0.032] | | -0.018 [-0.033; -0.004] | -0.030 [-0.036; -0.023] | | | -0.040 [-0.050; -0.030] | | | | -0.039 [-0.044; -0.034] | | |
| *Inefficient sleep* | -0.047 [-0.055; -0.039] | -0.045 [-0.052; -0.039] | | -0.027 [-0.035; -0.019] | -0.027 [-0.033; -0.021] | | | -0.045 [-0.051; -0.039] | | | | -0.039 [-0.043; -0.034] | | |
| **B. MAP/MARS** |  | |  |  | | |  | | | |  | | |  |
|  | **Word Learning**  Change/year [95% confidence interval] | | |  | | |  | | | |  | | |  |
|  |  |  |  |  | | |  | | | |  | | |  |
|  | **Males** | **Females** | |  | |  | | | |  | | |  |  |
| Sleep Health Score |  |  | |  | |  | | | |  | | |  |  |
| *0 poor sleep item (Ref.)* | -0.014 [-0.018; -0.009] | -0.017 [-0.020; -0.014] | |  | |  | | | |  | | |  |  |
| *1 poor sleep item* | -0.011 [-0.016; -0.005] | -0.015 [-0.018; -0.013] | |  | |  | | | |  | | |  |  |
| *2 poor sleep items* | -0.008 [-0.015; -0.001] | -0.011 [-0.014; -0.008] | |  | |  | | | |  | | |  |  |
| *3+ poor sleep items* | -0.016 [-0.025; -0.008] | -0.011 [-0.016; -0.006] | |  | |  | | | |  | | |  |  |
| Sleep health cluster |  |  | |  | |  | | | |  | | |  |  |
| *Average sleep (Ref.)* | -0.013 [-0.016; -0.009] | -0.016 [-0.019; -0.014] | |  | |  | | | |  | | |  |  |
| *Poor sleep* | -0.011 [-0.021; -0.002] | -0.010 [-0.017; -0.003] | |  | |  | | | |  | | |  |  |
| *Inefficient sleep* | -0.010 [-0.016; -0.004] | -0.011 [-0.014; -0.009] | |  | |  | | | |  | | |  |  |

Stratified results are only presented for those cognitive tests for which the interaction term was significant (i.e., p < 0.001). ^a^Indicates a difference in estimated sloop between males and females, i.e., confidence intervals of estimated means do not overlap horizontally. ^b^ Indicates a difference in estimated slope across levels of sleep health, i.e., confidence intervals of estimated means do not overlap vertically. Cognitive test scores were standardized and higher scores reflect better performance. Models were adjusted for age, race (MAP/MARS only), cohort (RS only), sex, education, smoking, alcohol, body mass index, and the number of comorbidities. MAP: Memory and Aging Project; MARS: Minority Aging Research Study.

## **Supplemental Table 8.** Associations of sleep health with cognition when additionally adjusting for depressive symptoms and medication.

| 1. **Rotterdam Study** |  | | | | | | | | |  |
| --- | --- | --- | --- | --- | --- | --- | --- | --- | --- | --- |
|  | **Word Learning** | **Substitution Task** | **Category Fluency** | **Stroop processing** | | **Stroop Interference** | |  |  |  |
| Total number of observations, *n* | 14327 | 14928 | 15010 | 14537 | | 14491 | |  |  |  |
|  | **Change/year [95% confidence interval]** | | | | | | | | |  |
| Sleep Health Score |  |  |  | |  | |  | |  |  |
| *0 poor sleep items (Ref.)* | -0.040 [-0.043; -0.037] | -0.051 [-0.053; -0.049] | -0.026 [-0.029; -0.024] | | -0.040 [-0.042; -0.038] | | -0.033 [-0.036; -0.031] | |  |  |
| *1 poor sleep item* | -0.041 [-0.045; -0.037] | -0.049 [-0.052; -0.046] | -0.023 [-0.027; -0.019] | | -0.042 [-0.045; -0.039] | | -0.032 [-0.035; -0.029] | |  |  |
| *2 poor sleep items* | -0.046 [-0.052; -0.040] | -0.050 [-0.054; -0.045] | -0.031 [-0.036; -0.025] | | -0.040 [-0.044; -0.036] | | -0.041 [-0.046; -0.036] | |  |  |
| *3+ poor sleep items* | -0.041 [-0.048; -0.034] | -0.048 [-0.053; -0.043] | -0.025 [-0.032; -0.018] | | -0.039 [-0.044; -0.034] | | -0.025 [-0.030; -0.019] | |  |  |
| Sleep health cluster |  |  |  | |  | |  | |  |  |
| *Average sleep (Ref.)* | -0.040 [-0.043; -0.038] | -0.051 [-0.052; -0.049] | -0.025 [-0.028; -0.023] | | -0.041 [-0.042; -0.039] | | -0.034 [-0.036; -0.032] | |  |  |
| *Poor sleep* | -0.040 [-0.046; -0.033] | -0.048 [-0.053; -0.044] | -0.027 [-0.033; -0.021] | | -0.039 [-0.044; -0.035] | | -0.028 [-0.033; -0.022] | |  |  |
| *Inefficient sleep* | -0.046 [-0.051; -0.041] | -0.049 [-0.053; -0.046] | -0.027 [-0.032; -0.022] | | -0.041 [-0.045; -0.038] | | -0.034 [-0.038; -0.030] | |  |  |
| **B. MAP/MARS** |  | | | | | | | | |  |
|  | **Word Learning** | **Substitution Task** | **Category Fluency** | | **Stroop processing** | | **Stroop interference** | | | |
| Total number of observations, *n* | 7805 | 7642 | 8993 | | 7571 | | 7576 | | | |
|  |  |  |  | |  | |  | | | |
| Sleep Health Score |  |  |  | |  | |  | | | |
| *0 poor sleep items (Ref.)* | -0.013 [-0.016; -0.011] | -0.028 [-0.030; -0.025] | -0.028 [-0.030; -0.025] | | -0.032 [-0.036; -0.028] | | -0.027 [-0.030; -0.024] | | | |
| *1 poor sleep item* | -0.012 [-0.015; -0.010] | -0.026 [-0.028; -0.023] | -0.026 [-0.028; -0.024] | | -0.029 [-0.032; -0.025] | | -0.027 [-0.029; -0.024] | | | |
| *2 poor sleep items* | -0.009 [-0.012; -0.006] | -0.024 [-0.027; -0.022] | **-0.021 [-0.023; -0.018]** | | -0.023 [-0.027; -0.019] | | -0.023 [-0.026; -0.020] | | | |
| *3+ poor sleep items* | -0.010 [-0.014; -0.006] | -0.021 [-0.025; -0.018] | -0.024 [-0.027; -0.020] | | -0.023 [-0.029; -0.017] | | -0.025 [-0.029; -0.021] | | | |
| Sleep health cluster |  |  |  | |  | |  | | | |
| *Average sleep (Ref.)* | -0.013 [-0.015; -0.011] | -0.026 [-0.028; -0.025] | -0.026 [-0.028; -0.025] | | -0.029 [-0.032; -0.026] | | -0.026 [-0.028; -0.024] | | | |
| *Poor sleep* | -0.010 [-0.015; -0.004] | -0.026 [-0.031; -0.021] | -0.023 [-0.028; -0.018] | | -0.026 [-0.034; -0.018] | | -0.027 [-0.033; -0.021] | | | |
| *Inefficient sleep* | -0.009 [-0.011; -0.007] | -0.024 [-0.026; -0.022] | -0.023 [-0.025; -0.021] | | -0.025 [-0.029; -0.022] | | -0.025 [-0.028; -0.023] | | | |

Effect sizes reflect the slope of the cognitive trajectory, i.e., the yearly change in cognitive score per year for each category. **Bolded** effect sizes reflect a slope that is significantly different from the reference category, i.e., p < 0.001 & confidence intervals do not overlap. Cognitive test scores were standardized and higher scores reflect better performance. Models were adjusted for age, cohort, sex, education, smoking, alcohol, body mass index, and the number of comorbidities. MAP: Memory and Aging Project; MARS: Minority Aging Research Study.

##

## **Supplemental Table 9.** Sleep health and risk of dementia.

| **A. Rotterdam Study** | 1148 Cases/ 83923.3 person-years |
| --- | --- |
| Sleep Health Score |  |
| *0 poor sleep items (Ref.)* | - |
| *1 poor sleep item* | 1.12 (0.98, 1.28) |
| *2 poor sleep items* | 1.06 (0.89, 1.26) |
| *3+ poor sleep items* | 0.93 (0.76, 1.13) |
|  |  |
| Sleep Health Score - *Continuous* | 0.99 (0.94, 1.04) |
|  |  |
| Sleep health cluster |  |
| *Average sleep (Ref.)* | - |
| *Inefficient sleep* | 0.93 (0.81, 1.08) |
| *Poor sleep* | - 1. 0.70, 1.01) |
| **B. MAP/MARS** | 286 Cases /7642.6 person-years |
| Sleep Health Score |  |
| *0 poor sleep items (Ref.)* | - |
| *1 poor sleep item* | 1.10 (0.81, 1.48) |
| *2 poor sleep items* | 1.24 (0.89, 1.71) |
| *3+ poor sleep items* | 1.08 (0.70, 1.65) |
|  |  |
| Sleep Health Score - *Continuous* | 1.04 (0.93, 1.16) |
|  |  |
| Sleep health cluster |  |
| *Average sleep (Ref.)* | - |
| *Inefficient sleep* | 1.13 (0.87, 1.46) |
| *Poor sleep* | 0.73 (0.42, 1.24) |

Effect estimates reflect Hazard Ratios with 95% Confidence Intervals. Models were adjusted for age, cohort, sex, education, smoking, alcohol, body mass index, and the number of comorbidities. MAP: Memory and Aging Project; MARS: Minority Aging Research Study.

## **Supplemental Table 10.** Sleep health and risk of dementia – Stratified by sex.

|  | **Males** | **Females** |
| --- | --- | --- |
| **A. Rotterdam Study** |  |  |
| Sleep Health Score |  |  |
| *0 poor sleep items (Ref.)* | - | - |
| *1 poor sleep item* | 0.97 (0.77, 1.22) | 1.19 (1.01, 1.41) |
| *2 poor sleep items* | 1.12 (0.82, 1.55) | 1.04 (0.83, 1.29) |
| *3+ poor sleep items* | 1.01 (0.65, 1.58) | 0.92 (0.74, 1.16) |
|  |  |  |
| Sleep Health Score - *Continuous* | 1.01 (0.91, 1.13) | 0.98 (0.92, 1.04) |
|  |  |  |
| Sleep health cluster |  |  |
| *Average sleep (Ref.)* | - | - |
| *Poor sleep* | 0.80 (0.51, 1.26) | 0.85 (0.69, 1.04) |
| *Inefficient sleep* | 0.99 (0.77, 1.28) | - 1. (0.76, 1.07) |
| **B. MAP/MARS** |  |  |
| Sleep Health Score |  |  |
| *0 poor sleep items (Ref.)* | - | - |
| *1 poor sleep item* | 0.52 (0.24, 1.13) | 1.24 (0.89, 1.75) |
| *2 poor sleep items* | 1.95 (0.99, 3.85) | 1.10 (0.75, 1.61) |
| *3+ poor sleep items* | 1.18 (0.41, 3.41) | 1.11 (0.69, 1.79) |
|  |  |  |
| Sleep Health Score - *Continuous* | 1.17 (0.89, 1.52) | 1.03 (0.91, 1.16) |
|  |  |  |
| Sleep health cluster |  |  |
| *Average sleep (Ref.)* | - | - |
| *Poor sleep* | 0.84 (0.28, 2.51) | 0.79 (0.43, 1.48) |
| *Inefficient sleep* | 1.30 (0.67, 2.54) | 1.09 (0.82, 1.44) |

Effect estimates reflect Hazard Ratios with 95% Confidence Intervals. Models were adjusted for age, race (MAP/MARS only), cohort (RS only), education, smoking, alcohol, body mass index, and the number of comorbidities. MAP: Memory and Aging Project; MARS: Minority Aging Research Study.

**Supplemental Figure 3.** Associations of sleep health with the risk of dementia, additionally adjusted for depressive symptoms and medication.

1.
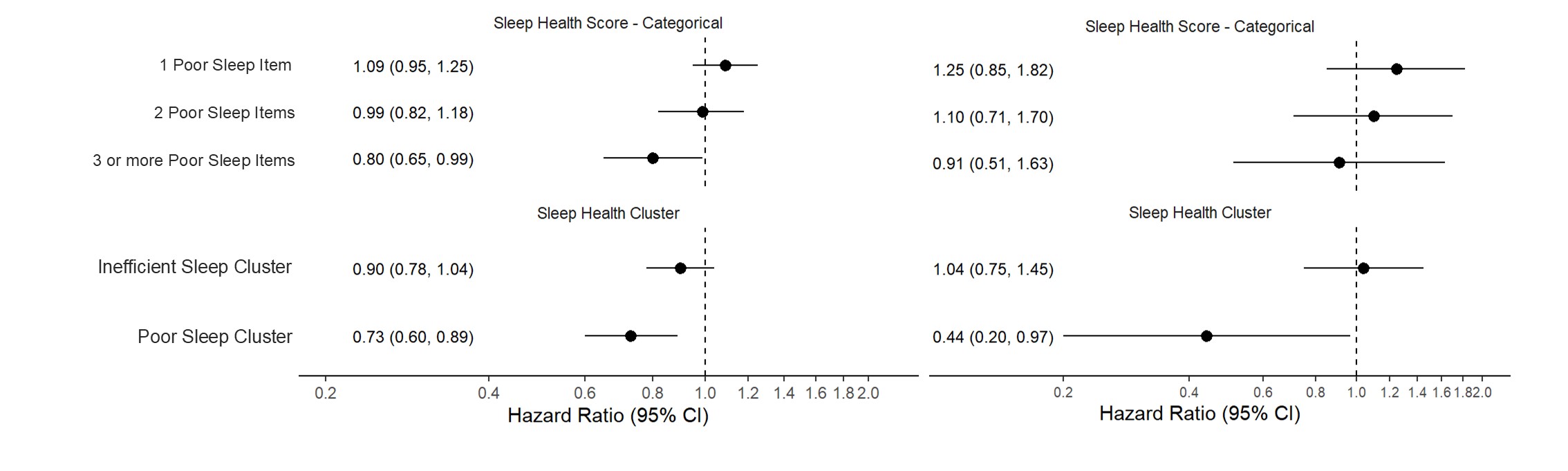
**Rotterdam Study B. MAP/MARS**

Effect estimates reflect the hazard ratio for dementia compared with the reference category (No poor sleep items/Average sleep cluster). Models were adjusted for age, race (MAP/MARS only), cohort (RS only), sex, education, smoking, alcohol, body mass index, and the number of comorbidities. MAP: Memory and Aging Project; MARS: Minority Aging Research Study.

**Supplemental Figure 4.** Associations of sleep health with the risk of Alzheimer’s disease.

1.
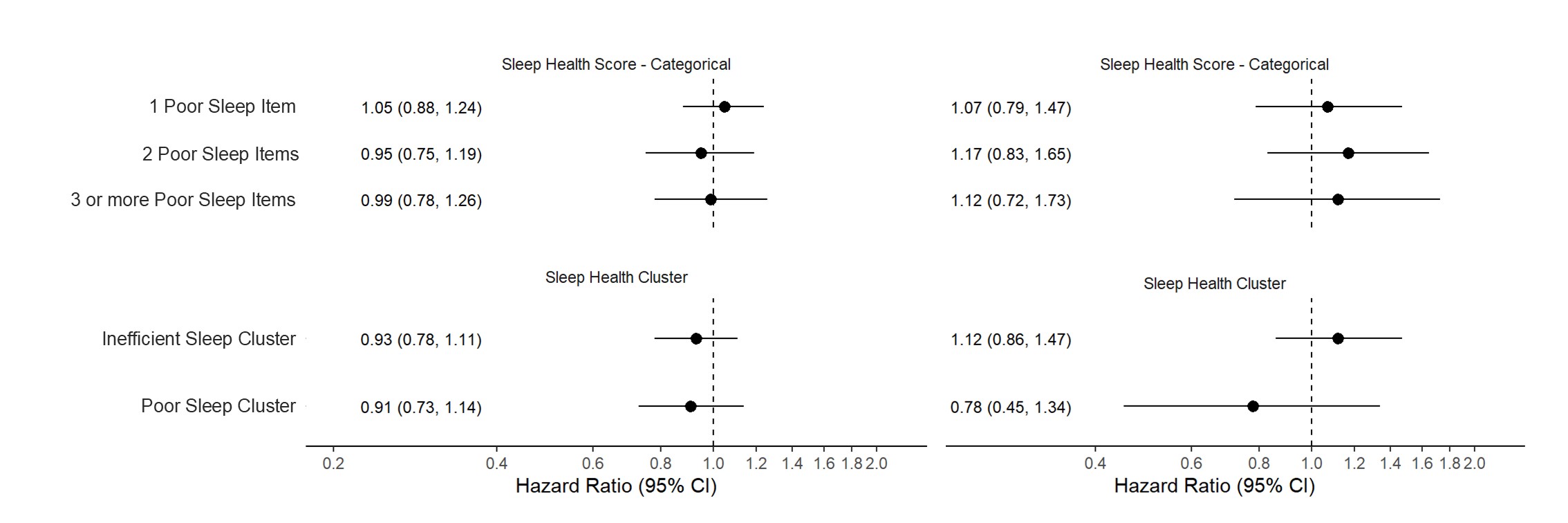
**Rotterdam Study** B. **MAP/MARS**

Effect estimates reflect the hazard ratio for dementia compared with the reference category (No poor sleep items/Average sleep cluster). Models were adjusted for age, race (MAP/MARS only), cohort (RS only), sex, education, smoking, alcohol, body mass index, and the number of comorbidities. MAP: Memory and Aging Project; MARS: Minority Aging Research Study.

## **Supplemental Figure 5**. Fine-Gray subdistribution hazard models.

1.
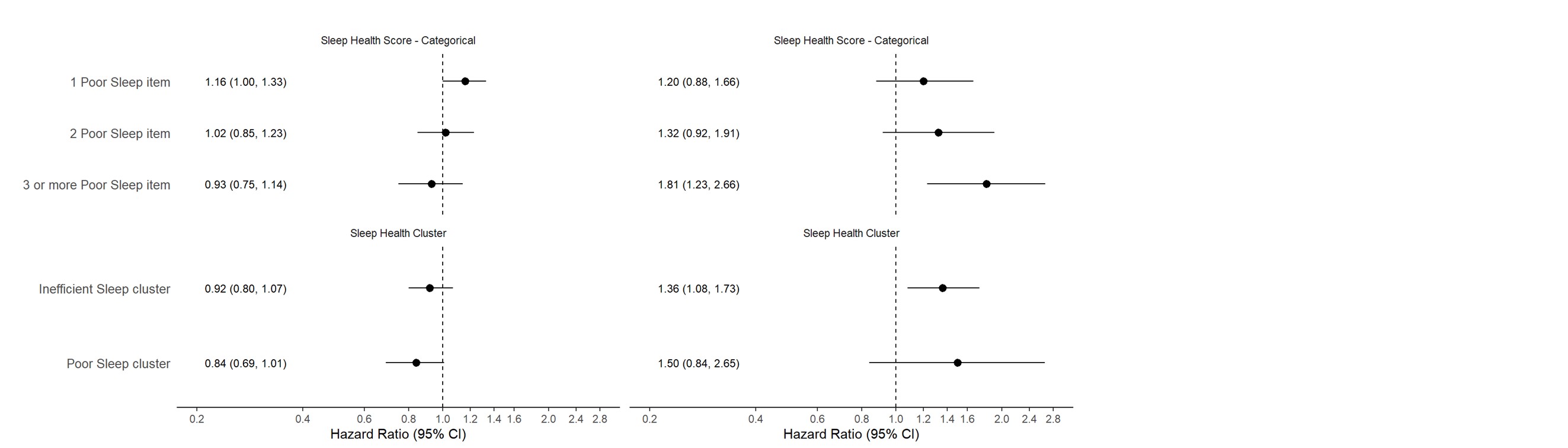
**Rotterdam Study B. MAP/MARS**

Effect estimates reflect the hazard ratio for dementia compared with the reference category (No poor sleep items/Average sleep cluster). Models were adjusted for age, race (MAP/MARS only), cohort (RS only), sex, education, smoking, alcohol, body mass index, and the number of comorbidities. MAP: Memory and Aging Project; MARS: Minority Aging Research Study.

##

## **Supplemental Table 11**. Sleep health and risk of dementia in Rotterdam Study – limited to participants > 75 years.

|  | **Cox proportional hazard models** | **Fine-Gray subdistribution hazard models** | |  |
| --- | --- | --- | --- | --- |
|  | 661 Cases/ 16 829 person-years | |  |  |
| Sleep Health Score |  |  | | |
| *0 poor sleep items (Ref.)* | - | - | | |
| *1 poor sleep item* | 0.97 (0.81, 1.16) | 1.03 (0.86, 1.24) | | |
| *2 poor sleep items* | 0.86 (0.68, 1.09) | 0.86 (0.68, 1.09) | | |
| *3+ poor sleep items* | 0.89 (0.69, 1.14) | 0.93 (0.73, 1.19) | | |
|  |  |  | | |
| Sleep health cluster |  |  | | |
| *Average sleep (Ref.)* | - | - | | |
| *Poor sleep* | 0.79 (0.66, 0.96) | 0.79 (0.65, 0.95) | | |
| *Inefficient sleep* | 0.77 (0.61, 0.98) | 0.80 (0.64, 1.01) | | |

Effect estimates reflect Hazard Ratios with 95% Confidence Intervals. Models were adjusted for age, cohort, sex, education, smoking, alcohol, body mass index, and the number of comorbidities.
